# Using DNA Barcodes to Identify Road-Killed Animals in Two Atlantic Forest Nature Reserves, Brazil

**DOI:** 10.1371/journal.pone.0134877

**Published:** 2015-08-05

**Authors:** Angélica H. Klippel, Pablo V. Oliveira, Karollini B. Britto, Bárbara F. Freire, Marcel R. Moreno, Alexandre R. dos Santos, Aureo Banhos, Greiciane G. Paneto

**Affiliations:** 1 Federal University of Espirito Santo, Centre of Agricultural Sciences, Alto Universitário, s/n, Guararema, Alegre, Espírito Santo, 29.500–000, Brazil; 2 Chico Mendes Institute of Biodiversity Conservation, Sooretama Biological Reserve, Highway BR-101, km 101, Linhares, Espírito Santo, 29.900–970, Brazil; Scientific Research Centre, Slovenian Academy of Sciences and Arts, SLOVENIA

## Abstract

Road mortality is the leading source of biodiversity loss in the world, especially due to fragmentation of natural habitats and loss of wildlife. The survey of the main species victims of roadkill is of fundamental importance for the better understanding of the problem, being necessary, for this, the correct species identification. The aim of this study was to verify if DNA barcodes can be applied to identify road-killed samples that often cannot be determined morphologically. For this purpose, 222 vertebrate samples were collected in a stretch of the BR-101 highway that crosses two Discovery Coast Atlantic Forest Natural Reserves, the Sooretama Biological Reserve and the Vale Natural Reserve, in Espírito Santo, Brazil. The mitochondrial COI gene was amplified, sequenced and confronted with the BOLD database. It was possible to identify 62.16% of samples, totaling 62 different species, including *Pyrrhura cruentata*, *Chaetomys subspinosus*, *Puma yagouaroundi* and *Leopardus wiedii* considered Vulnerable in the National Official List of Species of Endangered Wildlife. The most commonly identified animals were a bat (*Molossus molossus*), an opossum (*Didelphis aurita*) and a frog (*Trachycephalus mesophaeus*) species. Only one reptile was identified using the technique, probably due to lack of reference sequences in BOLD. These data may contribute to a better understanding of the impact of roads on species biodiversity loss and to introduce the DNA barcode technique to road ecology scenarios.

## Introduction

Road mortality has been one of the main causes of vertebrate species biodiversity loss [1,2]. It can reduce population abundance as wildlife–vehicle collisions add an extra toll to background mortality rates, reducing gene flow by eliminating either dispersing and/or breeding individuals [3]. Road-killed species are good representatives of the actual diversity of species of a particular site [4]. Genetic identification of multiple target species can also access road effects in species composition [5]. For those reasons, identification of these samples in road ecology studies is of great relevance, especially in natural reserves.

The Discovery Coast Atlantic Forest Reserves (DCAFR) are World Heritage Sites that consist of eight separate protected areas containing 112,000 ha of Atlantic forest and displays the biological richness and evolutionary history of the few remaining areas of Atlantic forest of Northeast Brazil, in Bahia and of Southeast Brazil, in Northern Espírito Santo [6]. Located in Espírito Santo are two DCARFs, the Sooretama Biological Reserve (SBR) and the Vale Natural Reserve (VNR) (also called Linhares Forest Reserve) which are protected areas of old Brazil, the conservation history of these areas dates from the 1940s [7]. These reserves form an Atlantic forest block measuring approximately 50,000 ha of table lands that since the late 1960s are crossed by a 25 kilometer stretch of federal highway called BR-101, fragmenting wildlife populations and causing high mortality by roadkill. Today, this highway is one of the most important and busiest in Brazil. There is a major concern about the potential loss of biodiversity due to the large amount of fauna and flora present in these reserves as well as about road expansion plans in the coming years.

The stretch that crosses the reserves has been monitored with some projects developed to evaluate the impact of this road stretch on the local fauna. However, a major obstacle found so far is that many animals are extremely disfigured after road-kills, hindering in many cases, their morphological identification.

Classical methods for species identification are based on morphological characteristics and depend on the knowledge of taxonomists who are usually experts in a particular group of organisms. Such professionals are difficult to find in today’s market [8,9] and many of the used methods require quality information (adult individuals with intact morphology) [10], which does not allow the identification of juveniles or fragmented exemplars. These factors represent obstacles to reliable species-level identification on a large scale and routinely performed. Another important issue is the existing high rates of biodiversity loss what makes necessary to accelerate the acquisition of knowledge regarding this topic [11] since many species will become extinct even before taxonomically recorded [12]. Although molecular methods may not replace traditional taxonomy, they can assist in solving this problem [13].

Knowing these conditions, DNA barcoding may be a powerful tool to solve or, at least, help on it. DNA barcodes uses, for animals, a specific region of the mitochondrial (mt) gene encoding cytochrome c oxidase subunit 1 (*COI*) to identify a species [14,15]. DNA barcodes from different species (deposited in museums or other institutions and previously identified by taxonomists) are being compiled in an online platform called *Barcode of Life Data Systems* (BOLD) (http://www.boldsystems.org/) [16] that promise to be the source of identification of all or most of the described species.

Within this context, the aim of this study was to verify whether DNA barcoding can be applied to identify road-killed samples.

## Materials and Methods

### Ethics statement

This study was approved by Instituto Chico Mendes de Conservação da Biodiversidade (ICMBio) in accordance with the Brazilian law (Permit Number: SISBIO31762-1).

### Study site and sample collection

**Fig 1:**
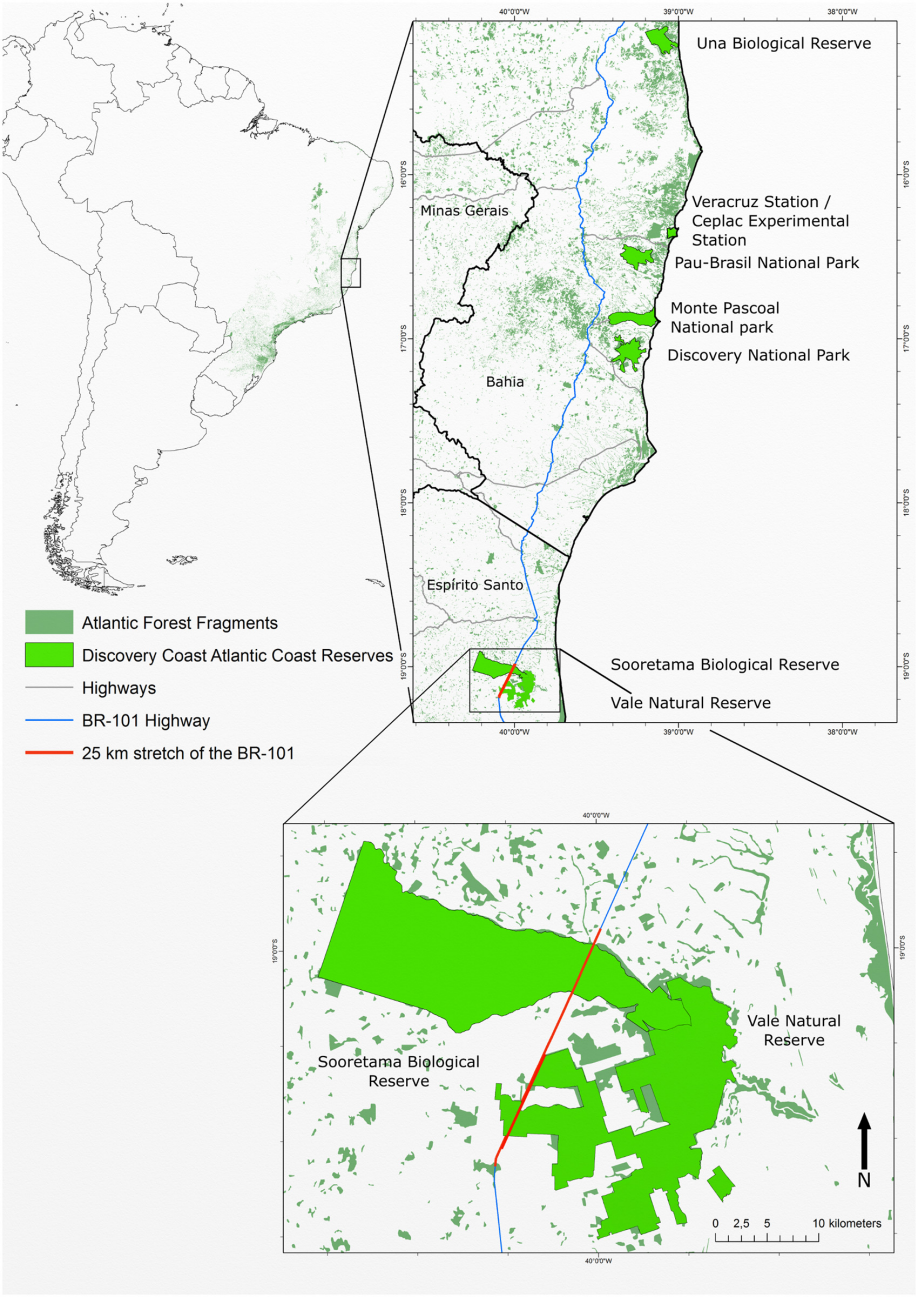
Map locating the Sooretama Biological Reserve and the Vale Natural Reserve in Southeast Brazil. Twenty-five kilometer stretch of BR 101 stretch of the BR-101 that intercepts SBR and VNR is showed in red double line. Note. Map generated with GRASS GIS, version 7.0. Layout generated with Inkscape vector graphics software, version 0.91. Sources: Instituto Brasileiro de Geografia e Estatística (IBGE); Departamento Nacional de Infraestrutura de Transportes (DNIT); Instituto Chico Mendes de Conservação da Biodiversidade (ICMBio); Reserva Natural Vale (RNV); United Nations Organization for Education, Science and Culture (UNESCO); United Nations Environment Programme's World Conservation Monitoring Centre (UNEP-WCMC).

SBR and VNR are located in Northern Espírito Santo State in South-Eastern Brazil, which is situated between 18°53'40'' and 19°15'20'' S latitude and 39°44'32'' and 40°16'51'' W longitude (Fig 1).

In this work, tissue samples from 222 road-killed animals were collected within the 25 kilometer stretch of the BR-101 that intercepts SBR and VNR, from April 2011 to July 2014. From these, 179 samples were part of known taxonomic groups (non-volant mammals, bats, birds, amphibians and reptiles) and 43 samples were from unknown taxonomic groups. Eighty-nine samples had photographic registry and 133 had no photo. Photos were used for previous morphological species identification and the results were compared to DNA barcoding identifications. These data are part of the roadkill monitoring held since 2010 on foot and by car. During monitoring, road-killed animals were registered and photographed, collection date and taxonomic group were noted. Tissue samples (skin and/or muscle) were collected and placed in a microtube with 70% alcohol and stored in a refrigerator (4°C) until analysis. Then, samples were deposited in a curated reference collection (Coleção de Tecidos Animais da Universidade Federal do Espírito Santo, reference number 2654–2838). When possible and in good condition, whole specimens were collected and stored. However, these specimens were not analyzed in this study because they were easily identified morphologically.

### DNA extraction, amplification and data processing

DNA was extracted from about 30 mg of animal tissue using NucleoSpin Tissue (Macherey-Nagel) and quantified using Nanodrop 2000 UV-Vis Spectrophotometer (Thermo Scientific). Primer pairs LCO1490 and HCO2198 were used for amplification of the mitochondrial cytochrome c oxidase I gene (COI) [17]. Alternative primers (BirdF1 and BirdR1; Chmf4 and Chmr4) were used when necessary [18,19]. PCR master mix was carried out using 8 μL ultrapure water, 1.25 μL of 10X Buffer PCR (Invitrogen Life Technologies), 2.5 mM MgCl_2_ (Invitrogen Life Technologies), 50 μM dNTPs, 100 nM of each primer and 1 Unit of Platinum Taq DNA polymerase (Invitrogen Life Technologies) making up a final volume of 12.5 μL mix for each sample. Samples were loaded onto Veriti thermo-cycler (Life Technologies). The thermal profile for all reactions consisted of 94°C for 1 minute, followed by 5 denaturation cycles at 94°C for 30 seconds, primer annealing at 45°C for 40 seconds, and extension at 72°C for 1 minute. Samples were submitted to 35 denaturation cycles at 94°C for 30 seconds, annealing of primers at 51°C for 40 seconds and extension at 72°C for 1 minute. A final cycle extension of 10 minutes at 72°C was included, followed by 4°C.

Negative controls were run in all PCR sets for possible reagent contamination. PCR products were electrophoresed through 1.2% agarose gel stained with ethidium bromide and visualized on an UV trans-illuminator. PCR products were enzymatically cleaned with ExoSap-IT (USB Corporation). Cleaned products were sequenced using BigDye Terminator v3.1 (Life Technologies) according to manufacturer’s protocols and analyzed using ABI 3500 Genetic Analyzer (Life Technologies).

### Sequence analyses

Electropherograms were generated in Sequence Analysis Software (Life Technologies) and edited using BioEdit Sequence Alignment Editor v7.0.5.3 [20]. Sequences were classified as “good quality electropherograms” when more than 90% of bases showed QV>20 and “poor quality electropherograms” when most of bases had QV<20. Good quality sequences were confronted with the BOLD platform (Barcode of Life Data system) using the option “Species Level Barcode Records”. At least 500 bp of COI gene and 99% of similarity were used for species identification. When less than 99% was obtained, a neighbor-joining (NJ) tree of K2P distances showing intra and interspecific variation was generated in BOLD and analyzed. In these cases, the sample was considered identified when assigned to a monophyletic group of sequences corresponding to a single species in the tree.

## Results

### Identification efficiency using DNA barcodes and BOLD

**Fig 2:**
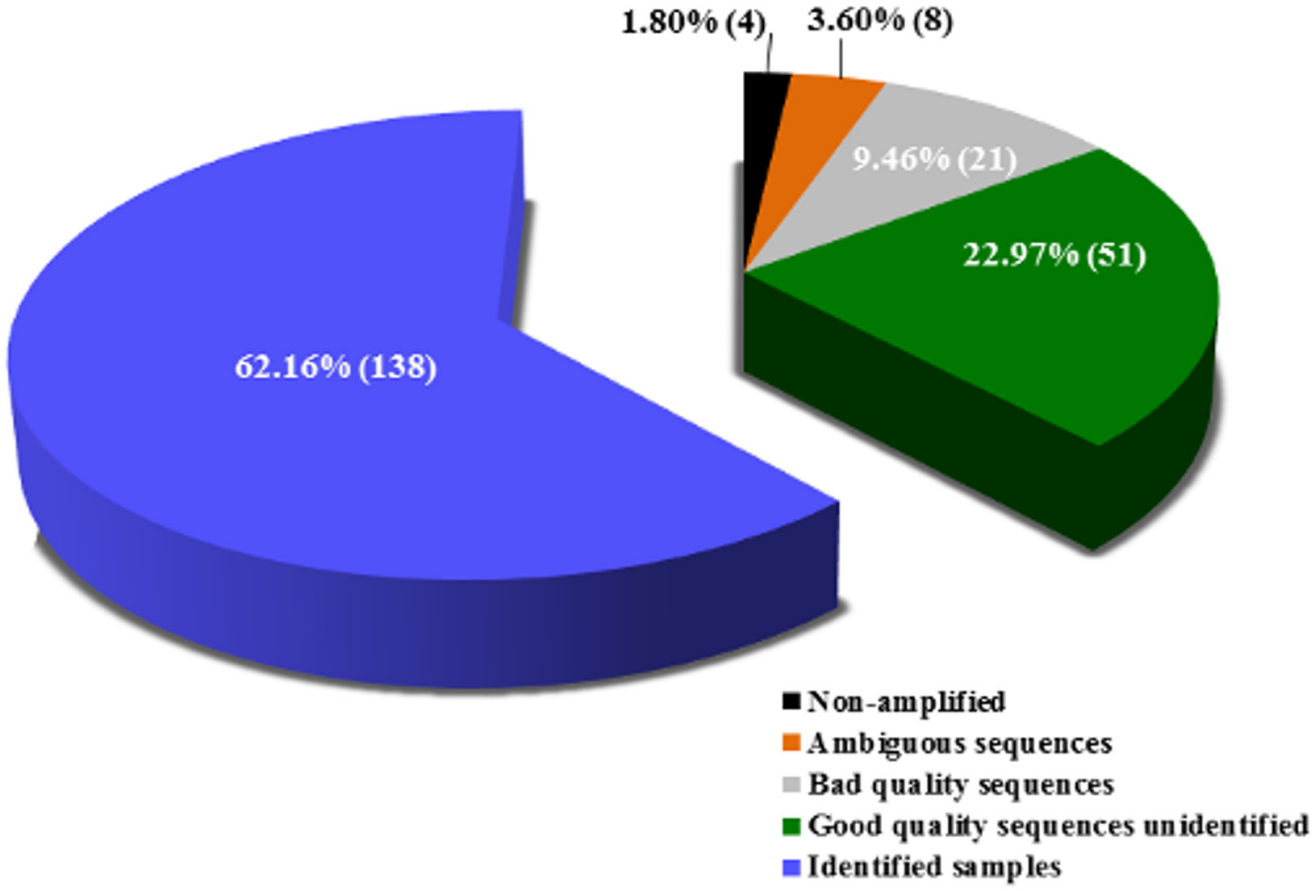
Percentage and absolute number of identified and unidentified samples in this study.

Of 222 tested samples, 138 (62.16%) could be identified using DNA barcoding and BOLD (Table 1). From these, 131 samples showed similarity >99% and seven had <99% in BOLD database. In the last case, the NJ tree was analyzed to consider the sample identified (Table 2). Eighty-four (37.84%) could not be identified at all: 51 (22.97%) samples generated good quality electropherograms, but they could not be identified in BOLD (“no match” was obtained, with exception of four samples with a match <99% but reproved in NJ tree analysis) (Table 2), 21 (9.46%) generated poor quality electropherograms even by repeating the technique, eight (3.60%) generated ambiguous identification and four (1.80%) did not amplify (Fig 2, Table 1 and S1 Table).

**Table 1:** Number of identified and unidentified species (and percentages) classified in taxonomic groups.

Taxonomic group	N° of samples	Identified samples (%)	Good quality sequence unidentified (%)	Bad quality seguence unidentified (%)	Ambiguous sequences	Non-amplified	N° of species identified
Non-volant mammals	40	35 (87.50%)	0 (0.00%)	3 (7.50%)	1 (2.50%)	1 (2.50%)	13
Bats	50	28 (56.00%)	11 (22.00%)	8 (16.00%)	2 (4.00%)	1 (2.00%)	14
Amphibians	21	14 (66.66%)	2 (9.52%)	3 (14.28%)	1 (4.76%)	1 (4.76%)	4
Birds	40	35 (87.50%)	1 (2.50%)	1 (2.50%)	3 (7.50%)	0 (0.00%)	25
Reptiles	28	0 (0.00%)	26 (92.86%)	2 (7.14%)	0 (0.00%)	0 (0.00%)	0
Unknown	43	26 (60.46%)	11 (25.58%)	4 (9.30%)	1 (2.32%)	1 (2.32%)	13*
**Total**	**222**	**138 (62.16%)**	**51 (22.97%)**	**21 (9.46%)**	**8 (3.60%)**	**4 (1.80%)**	**62**

*Six species identified exclusively in the Unknown taxonomic group

Where N° is the number

**Table 2:** Samples with less than 99% similarity after confronting sequences in BOLD.

**Taxonomic group**	**Species**	**Similarity in BOLD**	**Considered identified using NJ tree**	**Reference NJ tree**
Amphibian	*Scinax alter*	98.48%	Yes	(S1 Fig)
Bat	*Lampronycteris brachyotis*	98.26%	Yes	(S2 Fig)
Bat	*Lasiurus ega*	98.92%	No	(S3 Fig)
Bat	*Micronycteris minuta*	97.28%	Yes	(S4 Fig)
Bat	*Nyctinomops laticaudatus*	98.84%	No	(S5 Fig)
Bat	*Nyctinomops laticaudatus*	98.88%	No	(S6 Fig)
Bat	*Nyctinomops laticaudatus*	98.51%	No	(S7 Fig)
Bat	*Promops nasutus*	98.43%	Yes	(S8 Fig)
Bird	*Hemithraupis flavicollis*	97.65%	Yes	(S9 Fig)
Non-volant mammal	*Leopardus wiedii*	98.86%	Yes	(S10 Fig)
Reptile	*Oxybelis aeneus*	97.13%	Yes	(S11 Fig)

With regards to a prior taxonomic group classification, 35 (87.50%) of 40 samples of non-volant mammals were identified by DNA barcoding, 28 (56.00%) of 50 bats, 14 (66.67%) of 21 amphibians, 35 (87.50%) of 40 birds and 26 (60.46%) of 43 samples of unknown taxonomic groups. None of the 28 reptile (previously classified taxonomically) were identified using the technique described, although most samples (26 samples; 92.86%) exhibited good quality sequences (Table 1 & S1 Table).

### Identified species

The 138 samples identified in this study encompassed 62 different animal species: 13 non-volant mammals, 14 bats, four amphibians, 25 birds and six of previously taxonomic group unknown (one non-volant mammal, two amphibians, one bat, one bird and one reptile) (Table 1). The most commonly identified animals species were a bat (*Molossus molossus*), an opossum (*Didelphis aurita*) and a frog (*Trachycephalus mesophaeus*) species (Table 3).

**Table 3:** List of species, taxonomic group and number of samples identified using only DNA barcoding in this study.

Species—DNA Barcoding	Taxonomic group	Number of samples
*Aparasphenodon brunoi*	amphibian	4
*Hypsiboas faber*	amphibian	1
*Scinax alter*	amphibian/ ^a^	2
*Trachycephalus mesophaeus*	amphibian	12
*Hypsiboas semilineatus*	amphibian ^a^	1
*Leptodactylus natalensis*	amphibian ^a^	1
*Artibeus gnomus*	bat	1
*Carollia perspicillata*	bat	1
*Chiroderma villosum*	bat	2
*Lampronycteris brachyotis*	bat	1
*Lophostoma brasiliense*	bat	1
*Micronycteris minuta*	bat	1
*Molossus molossus*	bat	19
*Molossus rufus*	bat	1
*Myotis riparius*	bat	1
*Promops nasutus*	bat	2
*Rhinophylla pumilio*	bat	1
*Saccopteryx bilineata*	bat	2
*Trachops cirrhosus*	bat	1
*Vampyressa pusilla*	bat	1
*Anoura geoffroyi*	bat ^a^	1
*Buteo magnirostris*	bird	2
*Cairina moschata*	bird	1
*Coccyzus melacoryphus*	bird	1
*Coragyps atratus*	bird	1
*Crotophaga ani*	bird	1
*Dacnis cayana*	bird	1
*Dixiphia pipra*	bird	1
*Euphonia violacea*	bird	3
*Euphonia xanthogaster*	bird	1
*Hemithraupis flavicollis*	bird	1
*Lepidocolaptes squamatus*	bird	1
*Megascops choliba*	bird	1
*Myrmotherula axillaris*	bird	4
*Nyctidromus albicollis*	bird	1
*Pachyramphus polychopterus*	bird	2
*Patagioenas picazuro*	bird	1
*Piaya cayana*	bird	1
*Pipra rubrocapilla*	bird	4
*Porphyrio martinica*	bird	1
*Pteroglossus aracari*	bird	2
*Pyrrhura cruentata* *	bird	1
*Saltator maximus*	bird	2
*Tangara seledon*	bird	1
*Turdus leucomelas*	bird	1
*Vireo olivaceus*	bird	1
*Tapera naevia*	bird ^a^	1
*Bradypus variegatus*	non-volant mammals	1
*Callithrix geoffroyi*	non-volant mammals	3
*Cerdocyon thous*	non-volant mammals ^a^	1
*Chaetomys subspinosus* *	non-volant mammals	1
*Cuniculus paca*	non-volant mammals	6
*Dasypus septemcinctus*	non-volant mammals	1
*Didelphis aurita*	non-volant mammals	16
*Gracilinanus microtarsus*	non-volant mammals	2
*Marmosa murina*	non-volant mammals	2
*Marmosops incanus*	non-volant mammals	3
*Puma yagouaroundi*	non-volant mammals	1
*Sylvilagus brasiliensis*	non-volant mammals	2
*Leopardus wiedii* **	non-volant mammals ^a^	2
*Tamandua tetradactyla*	non-volant mammals ^a^	1
*Oxybelis aeneus*	reptile ^a^	1

^a^Taxonomic group previously unknown

* Vulnerable in IUCN Red List

** Near Threatened in IUCN Red List

### Ambiguous sequences

Ambiguous identification occurred in eight (3.6%) samples (Table 1), with BOLD reporting > 99% similarity to more than one species, thereby precluding the identification (Table 4).

**Table 4:** Ambiguous species identification obtained after BOLD analysis.

Species	Taxonomic group	Barcode Index Number Registry—BIN
*Artibeus lituratus*	Bats	AAA0874
*Artibeus intermedius*		
*Micronycteris microtis*	Bats	AAA6110
*Micronycteris megalotis*		
*Thamnophilus pelzelni*	Birds	AAW6887
*Thamnophilus ambiguus*		
*Thamnophilus pelzelni*	Birds	AAW6887
*Thamnophilus ambiguus*		
*Columbina talpacoti*	Birds	ACJ6362
*Columbina minuta*		
*Columbina buckleyi*		
*Tyrannus melancholicus*	Birds	AAB4120
*Myiodynastes maculatus*		
*Rhinella jimi*	Amphibians	no BIN published
*Rhinella schneideri*		
*Rhinella rubescens*		
*Rhinella marina*		
*Rhinella poeppigii*		
*Leptodactylus vastus*		
*Leptodactylus chaquensis*		
*Rattus rattus*	Non-volant mammals	AAB2207
*Rattus norvegicus*		

## Discussion

### Identified species

All 62 identified species were previously recorded in the studied area [21] or in the North of Espírito Santo State [22–31]. Therefore, the presence of all species confirmed in the region reinforces the molecular results.

The identified species *Pyrrhura cruentata and Chaetomys subspinosus* are considered Vulnerable– VU and the *Leopardus wiedii* is classified as Near Threatened—NT in the IUCN Red List of Threatened Species (2015). In Brazil, these three species and also *Puma yagouaroundi* are considered VU in the National Official List of Species of Endangered Wildlife [32]. This fact is very alarming since wildlife road-kill collaborates to the decline of populations making their recovery difficult, especially to endangered species [33,1]. It indicates the importance of road-killed species’ identification in DCAFRs, which was possible with the method applied in this study.

### Unidentified species

Some good quality DNA sequences (n = 51) could not be identified in BOLD demonstrating the absence of reference sequences for some species [34]. Most of the unidentified sequences were from reptiles (n = 28). Thus, most reptiles could not be identified in this study (with exception of one sample) due to the lack of reference sequences in BOLD. This was evidenced by the lack of studies covering this taxonomic group using COI gene [35]. The percentage of amphibian identification (66.66%) was also relatively low compared to the successful identification of non-volant mammals (87.50%) and bird samples (87.50%). The reason may be the fact that for reptiles and amphibians the 16S rDNA gene is more commonly used nowadays than COI, since the 16S fragment is considered superior to COI. In addition, it can be explained by methodological challenges caused by high mitochondrial DNA sequence variability, including PCR priming sites [36–38].

Despite this, some recent studies showed that COI is still a better marker for certain groups, such as species of salamanders [39]. Initiatives have been promoted to increase the number of reference sequences of reptiles and amphibian species in BOLD [35]. The identification of bats was also relatively low (56%), most of the sequences not identified in this group showed good quality electropherograms (n = 11; 22%). In spite of having a considerable amount of bat sequences deposited in BOLD [40,41], apparently it was not enough to identify all samples analyzed in this study.

Further efforts should be made to deposit sequences from specific biomes such as the Atlantic Forest, characterized by the presence of numerous endemic species and many others of restricted distributions [42,43]. In 2012, the National Research Council—CNPq founded the BrBol—Brazilian Network for Molecular Identification of Biodiversity, which aims to increase the number of COI sequences of neotropical species deposited in BOLD (brbol.org). This may be promising to improve the identification of roadkilled samples in the region.

### Ambiguous sequences

To verify the reasons for ambiguities, the BIN (Barcode Index Number) was checked. BIN is the result of an analysis method that applies clustering algorithms to distinguish partitions in the genetic distance among a group of individuals, creating a final array of OTUs (Operational Taxonomic Unit) that closely reflects species groupings. According to Ratnasingham & Hebert [44], cases of discordance between BIN assignments and current taxonomy reflect taxonomic errors, sequence contamination, the inability of sequence variation at COI to diagnose species because of introgression or their young age or deficits in Refined Single Linkage algorithms.

In this work, a bat sample was ambiguous to *Artibeus lituratus* and *Artibeus intermedius*. Clare et al. [40], for example, failed to distinguish these two species using COI gene in a study of 163 neotropical bat species. Another bat sample was also ambiguous between *Micronycteris megalotis* and *Micronycteris microtis*, and both occur in the region of this study. Clare et al. [40] distinguished these two species using COI, but did not find interspecific divergence between them and neither intraspecific divergence within *Micronycteris microtis*. However, *Micronycteris megalotis* presents substantial intraspecific divergent in mitochondrial lineages suggesting a cryptic species complex, but the regions of studied genes (COI and 7th intron region of the DBY gene on the Y-chromosome) show conflicting patterns of divergence and cannot exclude ongoing flow between intraspecific groups [45].

Two bird samples were ambiguous between *Thamnophilus pelzelni* and *Thamnophilus ambiguus*. Some morphological characteristics overlap considerably and may cause errors in identification. They are most easily distinguished by subtle and localized changes in plumage colors of males and females [46]. Lacerda et al. [47] suggest that the genetic divergence (using sequences of the Control Region, Cytochrome b and ND2 genes) found between *Thamnophilus ambiguus* and *Thamnophilus pelzelni* is high enough to corroborate the separate species status of these two antbird taxa. The geographical region studied is part of the distribution area of the *Thamnophilus ambiguus*, that is endemic to southeast Brazil and do not overlap the distribution area of the *Thamnophilus pelzelni*a [46]. Another bird was assigned as *Tyrannus melancholicus* and *Myiodynastes maculatus*, but it appears that the species is *Tyrannus melancholicus* since only one specimen of *M*. *maculates* is assigned in the BIN. The third bird sample was ambiguous among *Columbina talpacoti*, *Columbina minuta* and *Columbina buckleyi*.

One sample was ambiguous among *Rhinella jimi*, *Rhinella schneideri*, *Rhinella rubescens*, *Rhinella marina* and *Rhinella poeppigii*. However, only *Rhinella jimi* and *Rhinella schneideri* are known species in Espírito Santo [29].

One sample was ambiguous between *Rattus rattus* and *Rattus norvegicus*, although, it appears that the correct classification is *Rattus rattus* since only one specimen of *Rattus norvegicus* was assigned in the suggested BIN. At this point, it is important to highlight that *Rattus rattus* is an exotic species in the area what pinpoints the road as a route to the introduction of exotic species to protected areas.

### Low quality sequences and samples not amplified

Another relevant issue is that some samples (n = 21) had DNA amplified but generated low quality electropherograms and four did not amplify (even by repeating all the steps). It could be due to the exposure of biological material to climate action and repeated road-kills (more than once), compromising the quality of the material analyzed. It should be stressed that a 650-bp fragment of COI gene is difficult to amplify in degraded DNA samples [48]. However, we believe that the quality of sequences could be improved by a cautious protocol of collecting and storing samples, such as using 96% ethanol and below zero temperatures or using FTA Elute Card [49], and also those used for non-invasive sample collection [50]. The use of primer cocktails available [51] can increase the efficiency of barcode recovery and have demonstrated utility in a series of studies on different vertebrate groups [52,53]. A set of primers that amplify smaller fragments encompassing the entire region to be examined can also generate better results. On the other hand, it makes the work time consuming and laborious given that road-killed animal analysis involves a very wide range of taxonomic groups.

### Importance of the DNA barcoding technique to road-killed animal’s identification

Road-killed animal studies based on morphology identification have been often carried out in road ecology studies e.g., [54–56]. However, in many situations where animals were disfigured, it is not possible to carry out morphological identifications. Therefore, current DNA barcoding technique success seems to generate good results when compared to morphological identification.

**Fig 3:**
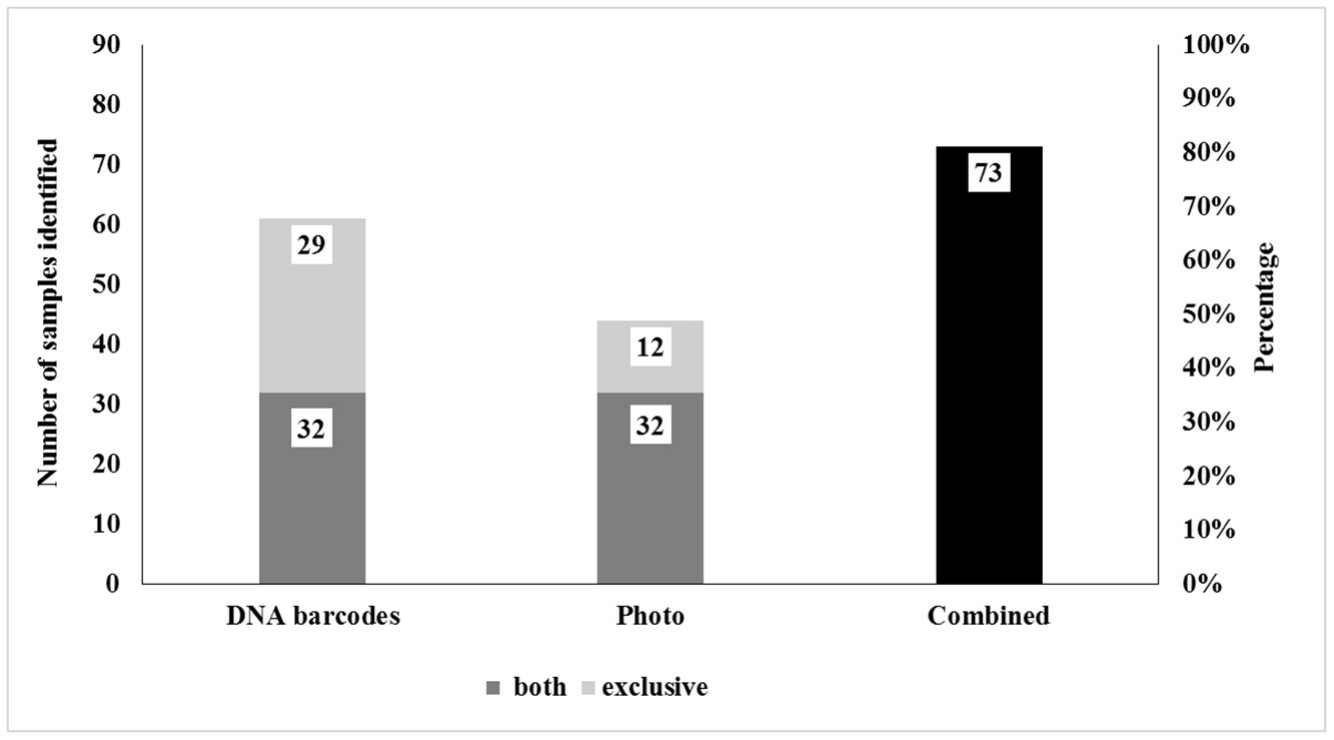
Number of samples and percentage of samples identified using DNA barcoding, photo identification and combined methods, respectively.

**Fig 4:**
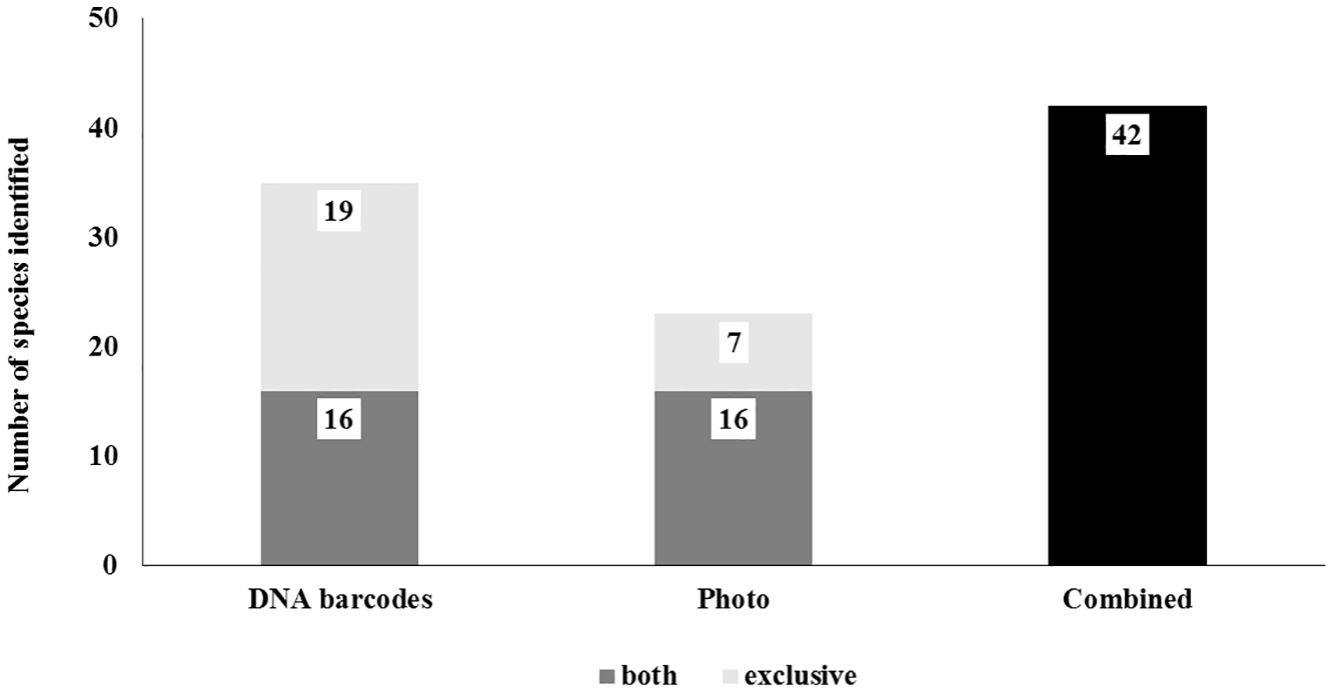
Number of species identified using DNA barcoding, photo identification and combined methods.

This study indicates that DNA barcodes can be used quite successfully as a complementary method to photo identification, at least in a restricted area. The number of species identified by photos (with the aid of guides and experts) was compared with DNA barcodes results from our samples. From 89 samples with photos, it was possible to identify only 44 (49%) with photos and 61 (69%) with DNA barcodes separately (Fig 3). A total of 35 different species were identified using DNA barcodes, while only 23 species were identified by photos. However, by combining both methods, DNA barcodes and photos, it was possible to identify 73 (82.02%) samples and 42 species (Figs 3 and 4).

From the eight species identified solely by photo, six had no reference sequences in BOLD: five of them generated good quality sequences (electropherograms), including three species of reptiles (*Epicrates cenchria*, *Pseudoboa nigra* and *Typhlops brongersmianus*), one of bat (*Promops nasutus*), one of bird (*Tinamus solitarius*), and one sample that generated poor quality sequence, a non-volant mammal (*Sphiggurus insidiosus*). Two species had their reference sequences deposited in BOLD but one sample generated a poor quality sequence, a bat (*Centronycteris maximiliani*), and three samples generated good quality sequences, another bat (*Nyctinomops laticaudatus*). However, although they kept 98.84, 98.88 and 98.51% of similarity to the species in BOLD, the identification was not confirmed using the NJ tree option in BOLD (Table 2). Thus, besides highlighting few shortcomings of reference sequences in BOLD, it was also confirmed the existence of gaps in the system, which does not allow the identification of many species (no match found), especially for reptiles.

Despite the current limitations in applying DNA barcodes, the results show that this method can be applied with relative success in road ecology studies. They can also provide important inputs to the work undertaken at Discovery Coast Atlantic Forest Reserves, which may serve to improve the understanding of the BR-101 highway impacts on species in this World Heritage Site. More importantly, identification of road-killed species can help to develop preventive measures focused on the animals most affected by road-kill and on endangered species.

### Data Accessibility

GenBank accession number: KR005651; KR017933 through KR017960; KT236170 through KT236278‏.

## Supporting Information

S1 Fig Neighbor-joining tree of K2P distances for *Scinax alter*. (PDF)

S2 Fig Neighbor-joining tree of K2P distances for *Lampronycteris brachyotis*. (PDF)

S3 Fig Neighbor-joining tree of K2P distances for *Lasiurus ega*. (PDF)

S4 Fig Neighbor-joining tree of K2P distances for *Micronycteris minuta*. (PDF)

S5 Fig Neighbor-joining tree of K2P distances for *Nyctinomops laticaudatus* (sample 1). (PDF)

S6 Fig Neighbor-joining tree of K2P distances for *Nyctinomops laticaudatus* (sample 2). (PDF)

S7 Fig Neighbor-joining tree of K2P distances for *Nyctinomops laticaudatus* (sample 3). (PDF)

S8 Fig Neighbor-joining tree of K2P distances for *Promops nasutus*. (PDF)

S9 Fig Neighbor-joining tree of K2P distances for *Hemithraupis flavicollis*. (PDF)

S10 Fig Neighbor-joining tree of K2P distances for *Leopardus wiedii*. (PDF)

S11 Fig Neighbor-joining tree of K2P distances for *Oxybelis aeneus*. (PDF)

S1 Table Information about 222 samples analyzed in this work (taxonomic group, species identified by DNA barcoding and species identified by photos). (DOC)
