# Supplementary material for: Using DNA Barcodes to Identify Road-Killed Animals in Two Atlantic Forest Nature Reserves, Brazil
Source: PLoS One. 2015 Aug 5;10(8):e0134877. doi: 10.1371/journal.pone.0134877 (PMC4526655; doi:10.1371/journal.pone.0134877)
Supplement: S1 Fig — (PDF) [file pone.0134877.s001.pdf]

BOLD TaxonID Tree

Title	:	COI SPECIES DATABASE Tree
Date	:	16-June-2015
Data Type	:	Nucleotide
Distance Model	:	Kimura 2 Parameter
Marker	:	COI-5P
Codon Positions	:	1st, 2nd, 3rd
Labels	:	Extra Info, Country & Province, Family
Filters	:	Length > 200
Attachment	:	Photographs & Spreadsheet

Sequence Count	:	100
Species count	:	27
Genus count	:	12
Family count	:	8
Unidentified	:	1

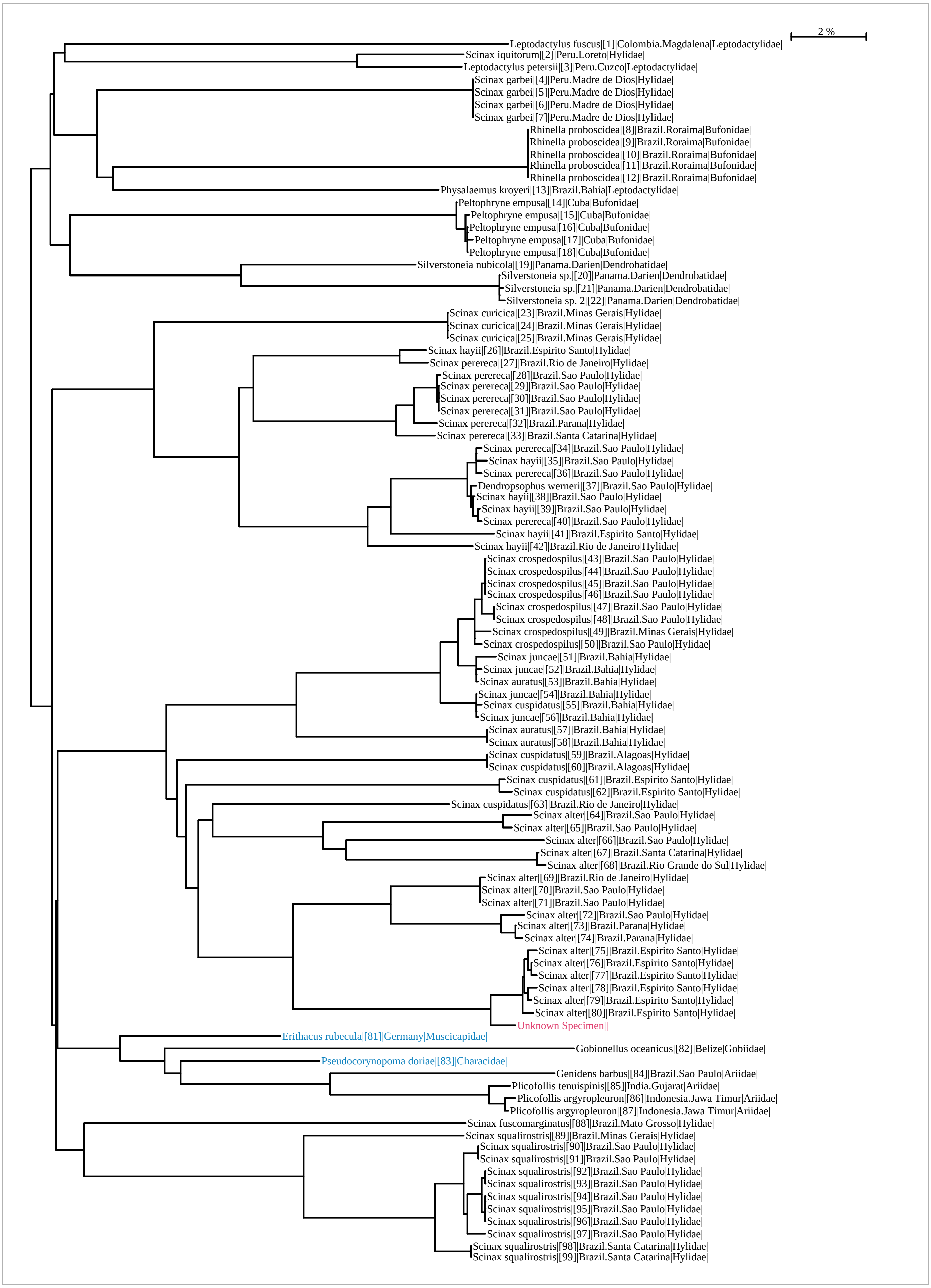
2 %
Leptodactylus fuscus|[1]|Colombia.Magdalena|Leptodactylidae|
Scinax iquitorum|[2]|Peru.Loreto|Hylidae|
Leptodactylus petersii|[3]|Peru.Cuzco|Leptodactylidae|
Scinax garbei|[4]|Peru.Madre de Dios|Hylidae|
Scinax garbei|[5]|Peru.Madre de Dios|Hylidae|
Scinax garbei|[6]|Peru.Madre de Dios|Hylidae|
Scinax garbei|[7]|Peru.Madre de Dios|Hylidae|
Rhinella proboscidea|[8]|Brazil.Roraima|Bufonidae|
Rhinella proboscidea|[9]|Brazil.Roraima|Bufonidae|
Rhinella proboscidea|[10]|Brazil.Roraima|Bufonidae|
Rhinella proboscidea|[11]|Brazil.Roraima|Bufonidae|
Rhinella proboscidea|[12]|Brazil.Roraima|Bufonidae|
Physalaemus kroyeri|[13]|Brazil.Bahia|Leptodactylidae|
Peltophryne empusa|[14]|Cuba|Bufonidae|
Peltophryne empusa|[15]|Cuba|Bufonidae|
Peltophryne empusa|[16]|Cuba|Bufonidae|
Peltophryne empusa|[17]|Cuba|Bufonidae|
Peltophryne empusa|[18]|Cuba|Bufonidae|
Silverstoneia nubicola|[19]|Panama.Darien|Dendrobatidae|
Silverstoneia sp.|[20]|Panama.Darien|Dendrobatidae|
Silverstoneia sp.|[21]|Panama.Darien|Dendrobatidae|
Silverstoneia sp. 2|[22]|Panama.Darien|Dendrobatidae|
Scinax curicica|[23]|Brazil.Minas Gerais|Hylidae|
Scinax curicica|[24]|Brazil.Minas Gerais|Hylidae|
Scinax curicica|[25]|Brazil.Minas Gerais|Hylidae|
Scinax hayii|[26]|Brazil.Espirito Santo|Hylidae|
Scinax perereca|[27]|Brazil.Rio de Janeiro|Hylidae|
Scinax perereca|[28]|Brazil.Sao Paulo|Hylidae|
Scinax perereca|[29]|Brazil.Sao Paulo|Hylidae|
Scinax perereca|[30]|Brazil.Sao Paulo|Hylidae|
Scinax perereca|[31]|Brazil.Sao Paulo|Hylidae|
Scinax perereca|[32]|Brazil.Parana|Hylidae|
Scinax perereca|[33]|Brazil.Santa Catarina|Hylidae|
Scinax perereca|[34]|Brazil.Sao Paulo|Hylidae|
Scinax hayii|[35]|Brazil.Sao Paulo|Hylidae|
Scinax perereca|[36]|Brazil.Sao Paulo|Hylidae|
Dendropsophus werneri|[37]|Brazil.Sao Paulo|Hylidae|
Scinax hayii|[38]|Brazil.Sao Paulo|Hylidae|
Scinax hayii|[39]|Brazil.Sao Paulo|Hylidae|
Scinax perereca|[40]|Brazil.Sao Paulo|Hylidae|
Scinax hayii|[41]|Brazil.Espirito Santo|Hylidae|
Scinax hayii|[42]|Brazil.Rio de Janeiro|Hylidae|
Scinax crospedospilus|[43]|Brazil.Sao Paulo|Hylidae|
Scinax crospedospilus|[44]|Brazil.Sao Paulo|Hylidae|
Scinax crospedospilus|[45]|Brazil.Sao Paulo|Hylidae|
Scinax crospedospilus|[46]|Brazil.Sao Paulo|Hylidae|
Scinax crospedospilus|[47]|Brazil.Sao Paulo|Hylidae|
Scinax crospedospilus|[48]|Brazil.Sao Paulo|Hylidae|
Scinax crospedospilus|[49]|Brazil.Minas Gerais|Hylidae|
Scinax crospedospilus|[50]|Brazil.Sao Paulo|Hylidae|
Scinax juncae|[51]|Brazil.Bahia|Hylidae|
Scinax juncae|[52]|Brazil.Bahia|Hylidae|
Scinax auratus|[53]|Brazil.Bahia|Hylidae|
Scinax juncae|[54]|Brazil.Bahia|Hylidae|
Scinax cuspidatus|[55]|Brazil.Bahia|Hylidae|
Scinax juncae|[56]|Brazil.Bahia|Hylidae|
Scinax auratus|[57]|Brazil.Bahia|Hylidae|
Scinax auratus|[58]|Brazil.Bahia|Hylidae|
Scinax cuspidatus|[59]|Brazil.Alagoas|Hylidae|
Scinax cuspidatus|[60]|Brazil.Alagoas|Hylidae|
Scinax cuspidatus|[61]|Brazil.Espirito Santo|Hylidae|
Scinax cuspidatus|[62]|Brazil.Espirito Santo|Hylidae|
Scinax cuspidatus|[63]|Brazil.Rio de Janeiro|Hylidae|
Scinax alter|[64]|Brazil.Sao Paulo|Hylidae|
Scinax alter|[65]|Brazil.Sao Paulo|Hylidae|
Scinax alter|[66]|Brazil.Sao Paulo|Hylidae|
Scinax alter|[67]|Brazil.Santa Catarina|Hylidae|
Scinax alter|[68]|Brazil.Rio Grande do Sul|Hylidae|
Scinax alter|[69]|Brazil.Rio de Janeiro|Hylidae|
Scinax alter|[70]|Brazil.Sao Paulo|Hylidae|
Scinax alter|[71]|Brazil.Sao Paulo|Hylidae|
Scinax alter|[72]|Brazil.Sao Paulo|Hylidae|
Scinax alter|[73]|Brazil.Parana|Hylidae|
Scinax alter|[74]|Brazil.Parana|Hylidae|
Scinax alter|[75]|Brazil.Espirito Santo|Hylidae|
Scinax alter|[76]|Brazil.Espirito Santo|Hylidae|
Scinax alter|[77]|Brazil.Espirito Santo|Hylidae|
Scinax alter|[78]|Brazil.Espirito Santo|Hylidae|
Scinax alter|[79]|Brazil.Espirito Santo|Hylidae|
Scinax alter|[80]|Brazil.Espirito Santo|Hylidae|
Unknown Specimen||
Erithacus rubecula|[81]|Germany|Muscicapidae|
Gobionellus oceanicus|[82]|Belize|Gobiidae|
Pseudocorynopoma doriae|[83]|Characidae|
Genidens barbus|[84]|Brazil.Sao Paulo|Ariidae|
Plicofollis tenuispinis|[85]|India.Gujarat|Ariidae|
Plicofollis argyropleuron|[86]|Indonesia.Jawa Timur|Ariidae|
Plicofollis argyropleuron|[87]|Indonesia.Jawa Timur|Ariidae|
Scinax fuscomarginatus|[88]|Brazil.Mato Grosso|Hylidae|
Scinax squalirostris|[89]|Brazil.Minas Gerais|Hylidae|
Scinax squalirostris|[90]|Brazil.Sao Paulo|Hylidae|
Scinax squalirostris|[91]|Brazil.Sao Paulo|Hylidae|
Scinax squalirostris|[92]|Brazil.Sao Paulo|Hylidae|
Scinax squalirostris|[93]|Brazil.Sao Paulo|Hylidae|
Scinax squalirostris|[94]|Brazil.Sao Paulo|Hylidae|
Scinax squalirostris|[95]|Brazil.Sao Paulo|Hylidae|
Scinax squalirostris|[96]|Brazil.Sao Paulo|Hylidae|
Scinax squalirostris|[97]|Brazil.Sao Paulo|Hylidae|
Scinax squalirostris|[98]|Brazil.Santa Catarina|Hylidae|
Scinax squalirostris|[99]|Brazil.Santa Catarina|Hylidae|
