# Supplementary material for: Using DNA Barcodes to Identify Road-Killed Animals in Two Atlantic Forest Nature Reserves, Brazil
Source: PLoS One. 2015 Aug 5;10(8):e0134877. doi: 10.1371/journal.pone.0134877 (PMC4526655; doi:10.1371/journal.pone.0134877)
Supplement: S2 Fig — (PDF) [file pone.0134877.s002.pdf]

BOLD TaxonID Tree

Title	: COI SPECIES DATABASE Tree
Date	: 16-June-2015
Data Type	: Nucleotide
Distance Model	: Kimura 2 Parameter
Marker	: COI-5P
Codon Positions	: 1st, 2nd, 3rd
Labels	: Extra Info, Country & Province, Family
Filters	: Length > 200
Attachment	: Photographs & Spreadsheet

Sequence Count	: 100
Species count	: 19
Genus count	: 9
Family count	: 7
Unidentified	: 1

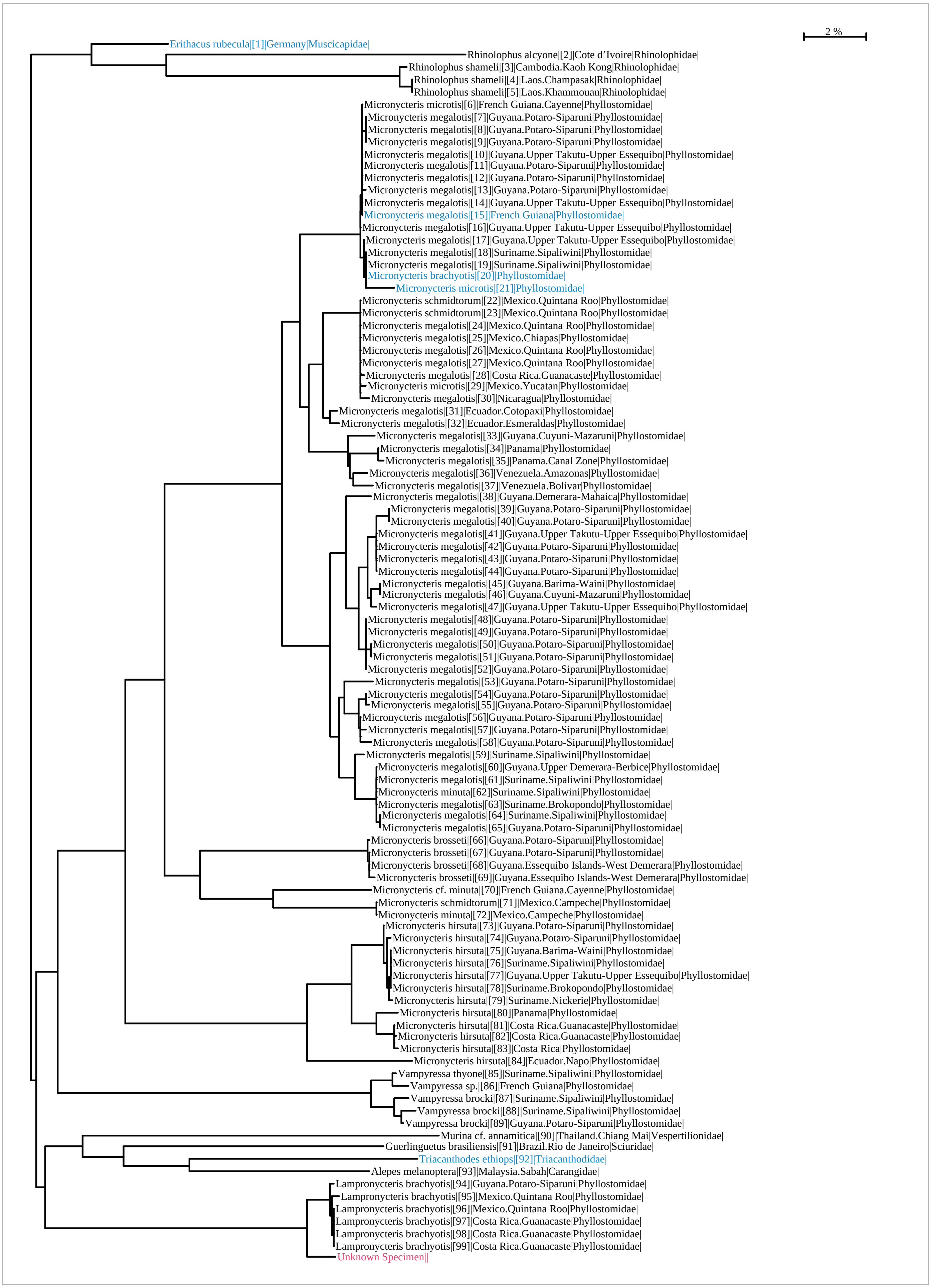
2 %
Erithacus rubecula|[1]|Germany|Muscicapidae|
Rhinolophus alcyone|[2]|Cote d'Ivoire|Rhinolophidae|
Rhinolophus shameli|[3]|Cambodia.Kaoh Kong|Rhinolophidae|
Rhinolophus shameli|[4]|Laos.Champasak|Rhinolophidae|
Rhinolophus shameli|[5]|Laos.Khammouan|Rhinolophidae|
Micronycteris microtis|[6]|French Guiana.Cayenne|Phyllostomidae|
Micronycteris megalotis|[7]|Guyana.Potaro-Siparuni|Phyllostomidae|
Micronycteris megalotis|[8]|Guyana.Potaro-Siparuni|Phyllostomidae|
Micronycteris megalotis|[9]|Guyana.Potaro-Siparuni|Phyllostomidae|
Micronycteris megalotis|[10]|Guyana.Upper Takutu-Upper Essequibo|Phyllostomidae|
Micronycteris megalotis|[11]|Guyana.Potaro-Siparuni|Phyllostomidae|
Micronycteris megalotis|[12]|Guyana.Potaro-Siparuni|Phyllostomidae|
Micronycteris megalotis|[13]|Guyana.Potaro-Siparuni|Phyllostomidae|
Micronycteris megalotis|[14]|Guyana.Upper Takutu-Upper Essequibo|Phyllostomidae|
Micronycteris megalotis|[15]|French Guiana|Phyllostomidae|
Micronycteris megalotis|[16]|Guyana.Upper Takutu-Upper Essequibo|Phyllostomidae|
Micronycteris megalotis|[17]|Guyana.Upper Takutu-Upper Essequibo|Phyllostomidae|
Micronycteris megalotis|[18]|Suriname.Sipaliwini|Phyllostomidae|
Micronycteris megalotis|[19]|Suriname.Sipaliwini|Phyllostomidae|
Micronycteris brachyotis|[20]|Phyllostomidae|
Micronycteris microtis|[21]|Phyllostomidae|
Micronycteris schmidtorum|[22]|Mexico.Quintana Roo|Phyllostomidae|
Micronycteris schmidtorum|[23]|Mexico.Quintana Roo|Phyllostomidae|
Micronycteris megalotis|[24]|Mexico.Quintana Roo|Phyllostomidae|
Micronycteris megalotis|[25]|Mexico.Chiapas|Phyllostomidae|
Micronycteris megalotis|[26]|Mexico.Quintana Roo|Phyllostomidae|
Micronycteris megalotis|[27]|Mexico.Quintana Roo|Phyllostomidae|
Micronycteris megalotis|[28]|Costa Rica.Guanacaste|Phyllostomidae|
Micronycteris microtis|[29]|Mexico.Yucatan|Phyllostomidae|
Micronycteris megalotis|[30]|Nicaragua|Phyllostomidae|
Micronycteris megalotis|[31]|Ecuador.Cotopaxi|Phyllostomidae|
Micronycteris megalotis|[32]|Ecuador.Esmeraldas|Phyllostomidae|
Micronycteris megalotis|[33]|Guyana.Cuyuni-Mazaruni|Phyllostomidae|
Micronycteris megalotis|[34]|Panama|Phyllostomidae|
Micronycteris megalotis|[35]|Panama.Canal Zone|Phyllostomidae|
Micronycteris megalotis|[36]|Venezuela.Amazonas|Phyllostomidae|
Micronycteris megalotis|[37]|Venezuela.Bolivar|Phyllostomidae|
Micronycteris megalotis|[38]|Guyana.Demerara-Mahaica|Phyllostomidae|
Micronycteris megalotis|[39]|Guyana.Potaro-Siparuni|Phyllostomidae|
Micronycteris megalotis|[40]|Guyana.Potaro-Siparuni|Phyllostomidae|
Micronycteris megalotis|[41]|Guyana.Upper Takutu-Upper Essequibo|Phyllostomidae|
Micronycteris megalotis|[42]|Guyana.Potaro-Siparuni|Phyllostomidae|
Micronycteris megalotis|[43]|Guyana.Potaro-Siparuni|Phyllostomidae|
Micronycteris megalotis|[44]|Guyana.Potaro-Siparuni|Phyllostomidae|
Micronycteris megalotis|[45]|Guyana.Barima-Waini|Phyllostomidae|
Micronycteris megalotis|[46]|Guyana.Cuyuni-Mazaruni|Phyllostomidae|
Micronycteris megalotis|[47]|Guyana.Upper Takutu-Upper Essequibo|Phyllostomidae|
Micronycteris megalotis|[48]|Guyana.Potaro-Siparuni|Phyllostomidae|
Micronycteris megalotis|[49]|Guyana.Potaro-Siparuni|Phyllostomidae|
Micronycteris megalotis|[50]|Guyana.Potaro-Siparuni|Phyllostomidae|
Micronycteris megalotis|[51]|Guyana.Potaro-Siparuni|Phyllostomidae|
Micronycteris megalotis|[52]|Guyana.Potaro-Siparuni|Phyllostomidae|
Micronycteris megalotis|[53]|Guyana.Potaro-Siparuni|Phyllostomidae|
Micronycteris megalotis|[54]|Guyana.Potaro-Siparuni|Phyllostomidae|
Micronycteris megalotis|[55]|Guyana.Potaro-Siparuni|Phyllostomidae|
Micronycteris megalotis|[56]|Guyana.Potaro-Siparuni|Phyllostomidae|
Micronycteris megalotis|[57]|Guyana.Potaro-Siparuni|Phyllostomidae|
Micronycteris megalotis|[58]|Guyana.Potaro-Siparuni|Phyllostomidae|
Micronycteris megalotis|[59]|Suriname.Sipaliwini|Phyllostomidae|
Micronycteris megalotis|[60]|Guyana.Upper Demerara-Berbice|Phyllostomidae|
Micronycteris megalotis|[61]|Suriname.Sipaliwini|Phyllostomidae|
Micronycteris minuta|[62]|Suriname.Sipaliwini|Phyllostomidae|
Micronycteris megalotis|[63]|Suriname.Brokopondo|Phyllostomidae|
Micronycteris megalotis|[64]|Suriname.Sipaliwini|Phyllostomidae|
Micronycteris megalotis|[65]|Guyana.Potaro-Siparuni|Phyllostomidae|
Micronycteris brosseti|[66]|Guyana.Potaro-Siparuni|Phyllostomidae|
Micronycteris brosseti|[67]|Guyana.Potaro-Siparuni|Phyllostomidae|
Micronycteris brosseti|[68]|Guyana.Essequibo Islands-West Demerara|Phyllostomidae|
Micronycteris brosseti|[69]|Guyana.Essequibo Islands-West Demerara|Phyllostomidae|
Micronycteris cf. minuta|[70]|French Guiana.Cayenne|Phyllostomidae|
Micronycteris schmidtorum|[71]|Mexico.Campeche|Phyllostomidae|
Micronycteris minuta|[72]|Mexico.Campeche|Phyllostomidae|
Micronycteris hirsuta|[73]|Guyana.Potaro-Siparuni|Phyllostomidae|
Micronycteris hirsuta|[74]|Guyana.Potaro-Siparuni|Phyllostomidae|
Micronycteris hirsuta|[75]|Guyana.Barima-Waini|Phyllostomidae|
Micronycteris hirsuta|[76]|Suriname.Sipaliwini|Phyllostomidae|
Micronycteris hirsuta|[77]|Guyana.Upper Takutu-Upper Essequibo|Phyllostomidae|
Micronycteris hirsuta|[78]|Suriname.Brokopondo|Phyllostomidae|
Micronycteris hirsuta|[79]|Suriname.Nickerie|Phyllostomidae|
Micronycteris hirsuta|[80]|Panama|Phyllostomidae|
Micronycteris hirsuta|[81]|Costa Rica.Guanacaste|Phyllostomidae|
Micronycteris hirsuta|[82]|Costa Rica.Guanacaste|Phyllostomidae|
Micronycteris hirsuta|[83]|Costa Rica|Phyllostomidae|
Micronycteris hirsuta|[84]|Ecuador.Napo|Phyllostomidae|
Vampyressa thyone|[85]|Suriname.Sipaliwini|Phyllostomidae|
Vampyressa sp.|[86]|French Guiana|Phyllostomidae|
Vampyressa brocki|[87]|Suriname.Sipaliwini|Phyllostomidae|
Vampyressa brocki|[88]|Suriname.Sipaliwini|Phyllostomidae|
Vampyressa brocki|[89]|Guyana.Potaro-Siparuni|Phyllostomidae|
Murina cf. annamitica|[90]|Thailand.Chiang Mai|Vespertilionidae|
Guerlinguetus brasiliensis|[91]|Brazil.Rio de Janeiro|Sciuridae|
Triacanthodes ethiops|[92]|Triacanthodidae|
Alepes melanoptera|[93]|Malaysia.Sabah|Carangidae|
Lampronycteris brachyotis|[94]|Guyana.Potaro-Siparuni|Phyllostomidae|
Lampronycteris brachyotis|[95]|Mexico.Quintana Roo|Phyllostomidae|
Lampronycteris brachyotis|[96]|Mexico.Quintana Roo|Phyllostomidae|
Lampronycteris brachyotis|[97]|Costa Rica.Guanacaste|Phyllostomidae|
Lampronycteris brachyotis|[98]|Costa Rica.Guanacaste|Phyllostomidae|
Lampronycteris brachyotis|[99]|Costa Rica.Guanacaste|Phyllostomidae|
Unknown Specimen||
