# Supplementary material for: Using DNA Barcodes to Identify Road-Killed Animals in Two Atlantic Forest Nature Reserves, Brazil
Source: PLoS One. 2015 Aug 5;10(8):e0134877. doi: 10.1371/journal.pone.0134877 (PMC4526655; doi:10.1371/journal.pone.0134877)
Supplement: S3 Fig — (PDF) [file pone.0134877.s003.pdf]

BOLD TaxonID Tree

Title	:	COI SPECIES DATABASE Tree
Date	:	16-June-2015
Data Type	:	Nucleotide
Distance Model	:	Kimura 2 Parameter
Marker	:	COI-5P
Codon Positions	:	1st, 2nd, 3rd
Labels	:	Extra Info, Country & Province, Family
Filters	:	Length > 200
Attachment	:	Photographs & Spreadsheet

Sequence Count	:	100
Species count	:	13
Genus count	:	7
Family count	:	1
Unidentified	:	1

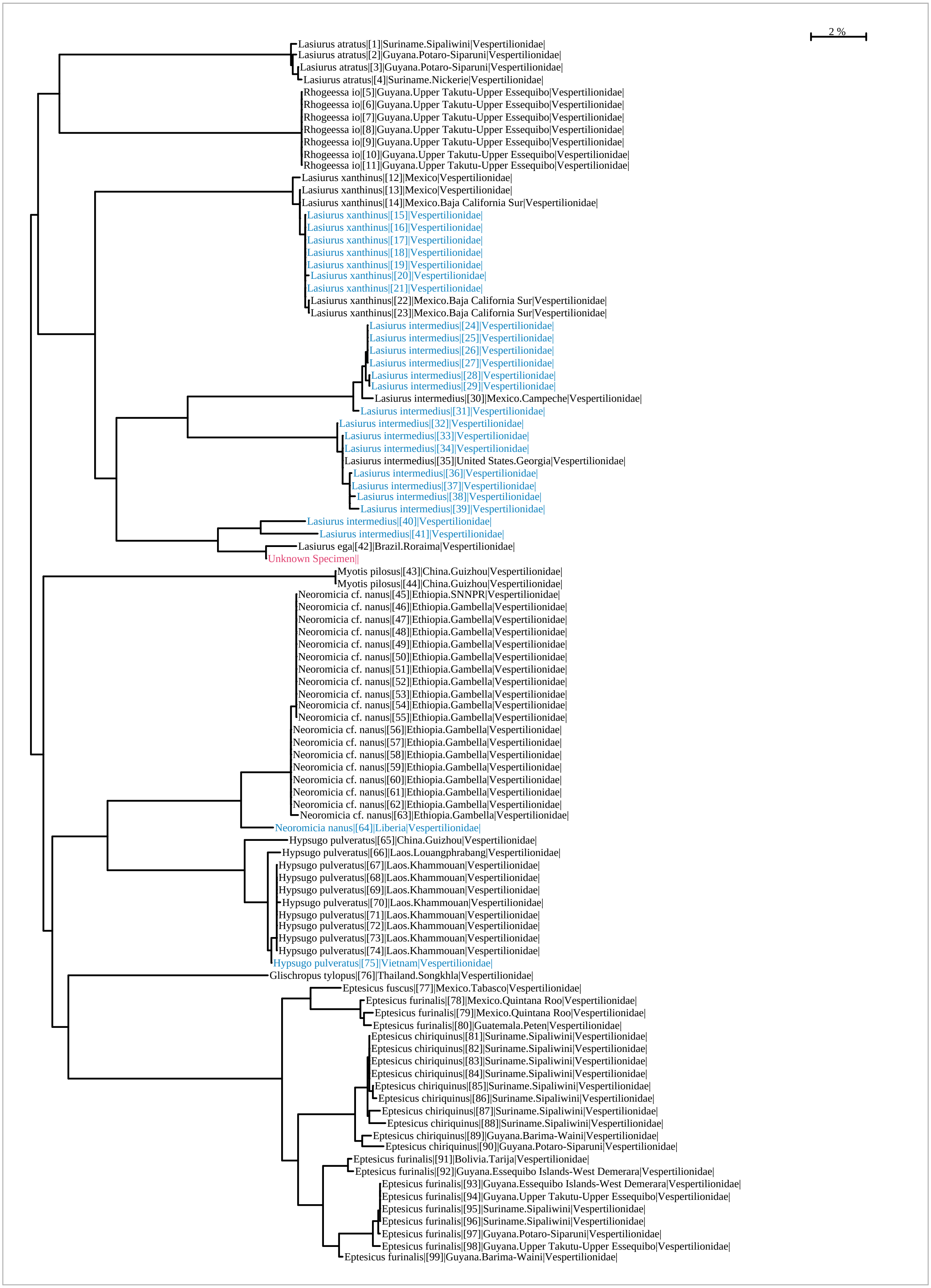
2 %
Lasiurus atratus|[1]|Suriname.Sipaliwini|Vespertilionidae|
Lasiurus atratus|[2]|Guyana.Potaro-Siparuni|Vespertilionidae|
Lasiurus atratus|[3]|Guyana.Potaro-Siparuni|Vespertilionidae|
Lasiurus atratus|[4]|Suriname.Nickerie|Vespertilionidae|
Rhogeessa io|[5]|Guyana.Upper Takutu-Upper Essequibo|Vespertilionidae|
Rhogeessa io|[6]|Guyana.Upper Takutu-Upper Essequibo|Vespertilionidae|
Rhogeessa io|[7]|Guyana.Upper Takutu-Upper Essequibo|Vespertilionidae|
Rhogeessa io|[8]|Guyana.Upper Takutu-Upper Essequibo|Vespertilionidae|
Rhogeessa io|[9]|Guyana.Upper Takutu-Upper Essequibo|Vespertilionidae|
Rhogeessa io|[10]|Guyana.Upper Takutu-Upper Essequibo|Vespertilionidae|
Rhogeessa io|[11]|Guyana.Upper Takutu-Upper Essequibo|Vespertilionidae|
Lasiurus xanthinus|[12]|Mexico|Vespertilionidae|
Lasiurus xanthinus|[13]|Mexico|Vespertilionidae|
Lasiurus xanthinus|[14]|Mexico.Baja California Sur|Vespertilionidae|
Lasiurus xanthinus|[15]|Vespertilionidae|
Lasiurus xanthinus|[16]|Vespertilionidae|
Lasiurus xanthinus|[17]|Vespertilionidae|
Lasiurus xanthinus|[18]|Vespertilionidae|
Lasiurus xanthinus|[19]|Vespertilionidae|
Lasiurus xanthinus|[20]|Vespertilionidae|
Lasiurus xanthinus|[21]|Vespertilionidae|
Lasiurus xanthinus|[22]|Mexico.Baja California Sur|Vespertilionidae|
Lasiurus xanthinus|[23]|Mexico.Baja California Sur|Vespertilionidae|
Lasiurus intermedius|[24]|Vespertilionidae|
Lasiurus intermedius|[25]|Vespertilionidae|
Lasiurus intermedius|[26]|Vespertilionidae|
Lasiurus intermedius|[27]|Vespertilionidae|
Lasiurus intermedius|[28]|Vespertilionidae|
Lasiurus intermedius|[29]|Vespertilionidae|
Lasiurus intermedius|[30]|Mexico.Campeche|Vespertilionidae|
Lasiurus intermedius|[31]|Vespertilionidae|
Lasiurus intermedius|[32]|Vespertilionidae|
Lasiurus intermedius|[33]|Vespertilionidae|
Lasiurus intermedius|[34]|Vespertilionidae|
Lasiurus intermedius|[35]|United States.Georgia|Vespertilionidae|
Lasiurus intermedius|[36]|Vespertilionidae|
Lasiurus intermedius|[37]|Vespertilionidae|
Lasiurus intermedius|[38]|Vespertilionidae|
Lasiurus intermedius|[39]|Vespertilionidae|
Lasiurus intermedius|[40]|Vespertilionidae|
Lasiurus intermedius|[41]|Vespertilionidae|
Lasiurus ega|[42]|Brazil.Roraima|Vespertilionidae|
Unknown Specimen||
Myotis pilosus|[43]|China.Guizhou|Vespertilionidae|
Myotis pilosus|[44]|China.Guizhou|Vespertilionidae|
Neoromicia cf. nanus|[45]|Ethiopia.SNNPR|Vespertilionidae|
Neoromicia cf. nanus|[46]|Ethiopia.Gambella|Vespertilionidae|
Neoromicia cf. nanus|[47]|Ethiopia.Gambella|Vespertilionidae|
Neoromicia cf. nanus|[48]|Ethiopia.Gambella|Vespertilionidae|
Neoromicia cf. nanus|[49]|Ethiopia.Gambella|Vespertilionidae|
Neoromicia cf. nanus|[50]|Ethiopia.Gambella|Vespertilionidae|
Neoromicia cf. nanus|[51]|Ethiopia.Gambella|Vespertilionidae|
Neoromicia cf. nanus|[52]|Ethiopia.Gambella|Vespertilionidae|
Neoromicia cf. nanus|[53]|Ethiopia.Gambella|Vespertilionidae|
Neoromicia cf. nanus|[54]|Ethiopia.Gambella|Vespertilionidae|
Neoromicia cf. nanus|[55]|Ethiopia.Gambella|Vespertilionidae|
Neoromicia cf. nanus|[56]|Ethiopia.Gambella|Vespertilionidae|
Neoromicia cf. nanus|[57]|Ethiopia.Gambella|Vespertilionidae|
Neoromicia cf. nanus|[58]|Ethiopia.Gambella|Vespertilionidae|
Neoromicia cf. nanus|[59]|Ethiopia.Gambella|Vespertilionidae|
Neoromicia cf. nanus|[60]|Ethiopia.Gambella|Vespertilionidae|
Neoromicia cf. nanus|[61]|Ethiopia.Gambella|Vespertilionidae|
Neoromicia cf. nanus|[62]|Ethiopia.Gambella|Vespertilionidae|
Neoromicia cf. nanus|[63]|Ethiopia.Gambella|Vespertilionidae|
Neoromicia nanus|[64]|Liberia|Vespertilionidae|
Hypsugo pulveratus|[65]|China.Guizhou|Vespertilionidae|
Hypsugo pulveratus|[66]|Laos.Louangphrabang|Vespertilionidae|
Hypsugo pulveratus|[67]|Laos.Khammouan|Vespertilionidae|
Hypsugo pulveratus|[68]|Laos.Khammouan|Vespertilionidae|
Hypsugo pulveratus|[69]|Laos.Khammouan|Vespertilionidae|
Hypsugo pulveratus|[70]|Laos.Khammouan|Vespertilionidae|
Hypsugo pulveratus|[71]|Laos.Khammouan|Vespertilionidae|
Hypsugo pulveratus|[72]|Laos.Khammouan|Vespertilionidae|
Hypsugo pulveratus|[73]|Laos.Khammouan|Vespertilionidae|
Hypsugo pulveratus|[74]|Laos.Khammouan|Vespertilionidae|
Hypsugo pulveratus|[75]|Vietnam|Vespertilionidae|
Glischropus tylopus|[76]|Thailand.Songkhla|Vespertilionidae|
Eptesicus fuscus|[77]|Mexico.Tabasco|Vespertilionidae|
Eptesicus furinalis|[78]|Mexico.Quintana Roo|Vespertilionidae|
Eptesicus furinalis|[79]|Mexico.Quintana Roo|Vespertilionidae|
Eptesicus furinalis|[80]|Guatemala.Peten|Vespertilionidae|
Eptesicus chiriquinus|[81]|Suriname.Sipaliwini|Vespertilionidae|
Eptesicus chiriquinus|[82]|Suriname.Sipaliwini|Vespertilionidae|
Eptesicus chiriquinus|[83]|Suriname.Sipaliwini|Vespertilionidae|
Eptesicus chiriquinus|[84]|Suriname.Sipaliwini|Vespertilionidae|
Eptesicus chiriquinus|[85]|Suriname.Sipaliwini|Vespertilionidae|
Eptesicus chiriquinus|[86]|Suriname.Sipaliwini|Vespertilionidae|
Eptesicus chiriquinus|[87]|Suriname.Sipaliwini|Vespertilionidae|
Eptesicus chiriquinus|[88]|Suriname.Sipaliwini|Vespertilionidae|
Eptesicus chiriquinus|[89]|Guyana.Barima-Waini|Vespertilionidae|
Eptesicus chiriquinus|[90]|Guyana.Potaro-Siparuni|Vespertilionidae|
Eptesicus furinalis|[91]|Bolivia.Tarija|Vespertilionidae|
Eptesicus furinalis|[92]|Guyana.Essequibo Islands-West Demerara|Vespertilionidae|
Eptesicus furinalis|[93]|Guyana.Essequibo Islands-West Demerara|Vespertilionidae|
Eptesicus furinalis|[94]|Guyana.Upper Takutu-Upper Essequibo|Vespertilionidae|
Eptesicus furinalis|[95]|Suriname.Sipaliwini|Vespertilionidae|
Eptesicus furinalis|[96]|Suriname.Sipaliwini|Vespertilionidae|
Eptesicus furinalis|[97]|Guyana.Potaro-Siparuni|Vespertilionidae|
Eptesicus furinalis|[98]|Guyana.Upper Takutu-Upper Essequibo|Vespertilionidae|
Eptesicus furinalis|[99]|Guyana.Barima-Waini|Vespertilionidae|
