# Supplementary material for: Using DNA Barcodes to Identify Road-Killed Animals in Two Atlantic Forest Nature Reserves, Brazil
Source: PLoS One. 2015 Aug 5;10(8):e0134877. doi: 10.1371/journal.pone.0134877 (PMC4526655; doi:10.1371/journal.pone.0134877)
Supplement: S4 Fig — (PDF) [file pone.0134877.s004.pdf]

BOLD TaxonID Tree

Title	:	COI SPECIES DATABASE Tree
Date	:	16-June-2015
Data Type	:	Nucleotide
Distance Model	:	Kimura 2 Parameter
Marker	:	COI-5P
Codon Positions	:	1st, 2nd, 3rd
Labels	:	Extra Info, Country & Province, Family
Filters	:	Length > 200
Attachment	:	Photographs & Spreadsheet

Sequence Count	:	100
Species count	:	18
Genus count	:	11
Family count	:	6
Unidentified	:	1

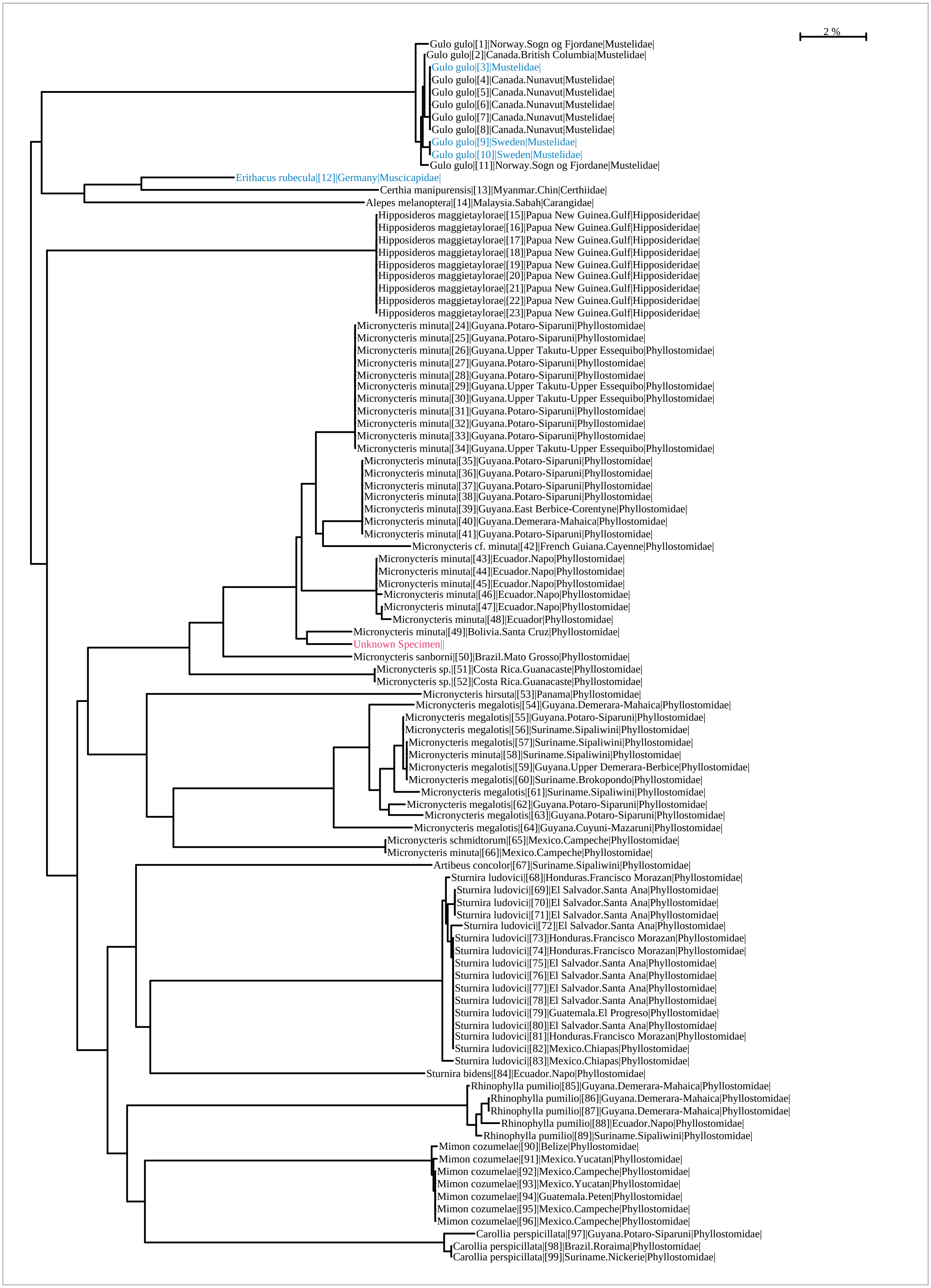
2 %
Gulo gulo|[1]|Norway.Sogn og Fjordane|Mustelidae|
Gulo gulo|[2]|Canada.British Columbia|Mustelidae|
Gulo gulo|[3]|Mustelidae|
Gulo gulo|[4]|Canada.Nunavut|Mustelidae|
Gulo gulo|[5]|Canada.Nunavut|Mustelidae|
Gulo gulo|[6]|Canada.Nunavut|Mustelidae|
Gulo gulo|[7]|Canada.Nunavut|Mustelidae|
Gulo gulo|[8]|Canada.Nunavut|Mustelidae|
Gulo gulo|[9]|Sweden|Mustelidae|
Gulo gulo|[10]|Sweden|Mustelidae|
Gulo gulo|[11]|Norway.Sogn og Fjordane|Mustelidae|
Erithacus rubecula|[12]|Germany|Muscicapidae|
Certhia manipurensis|[13]|Myanmar.Chin|Certhiidae|
Alepes melanoptera|[14]|Malaysia.Sabah|Carangidae|
Hipposideros maggietaylorae|[15]|Papua New Guinea.Gulf|Hipposideridae|
Hipposideros maggietaylorae|[16]|Papua New Guinea.Gulf|Hipposideridae|
Hipposideros maggietaylorae|[17]|Papua New Guinea.Gulf|Hipposideridae|
Hipposideros maggietaylorae|[18]|Papua New Guinea.Gulf|Hipposideridae|
Hipposideros maggietaylorae|[19]|Papua New Guinea.Gulf|Hipposideridae|
Hipposideros maggietaylorae|[20]|Papua New Guinea.Gulf|Hipposideridae|
Hipposideros maggietaylorae|[21]|Papua New Guinea.Gulf|Hipposideridae|
Hipposideros maggietaylorae|[22]|Papua New Guinea.Gulf|Hipposideridae|
Hipposideros maggietaylorae|[23]|Papua New Guinea.Gulf|Hipposideridae|
Micronycteris minuta|[24]|Guyana.Potaro-Siparuni|Phyllostomidae|
Micronycteris minuta|[25]|Guyana.Potaro-Siparuni|Phyllostomidae|
Micronycteris minuta|[26]|Guyana.Upper Takutu-Upper Essequibo|Phyllostomidae|
Micronycteris minuta|[27]|Guyana.Potaro-Siparuni|Phyllostomidae|
Micronycteris minuta|[28]|Guyana.Potaro-Siparuni|Phyllostomidae|
Micronycteris minuta|[29]|Guyana.Upper Takutu-Upper Essequibo|Phyllostomidae|
Micronycteris minuta|[30]|Guyana.Upper Takutu-Upper Essequibo|Phyllostomidae|
Micronycteris minuta|[31]|Guyana.Potaro-Siparuni|Phyllostomidae|
Micronycteris minuta|[32]|Guyana.Potaro-Siparuni|Phyllostomidae|
Micronycteris minuta|[33]|Guyana.Potaro-Siparuni|Phyllostomidae|
Micronycteris minuta|[34]|Guyana.Upper Takutu-Upper Essequibo|Phyllostomidae|
Micronycteris minuta|[35]|Guyana.Potaro-Siparuni|Phyllostomidae|
Micronycteris minuta|[36]|Guyana.Potaro-Siparuni|Phyllostomidae|
Micronycteris minuta|[37]|Guyana.Potaro-Siparuni|Phyllostomidae|
Micronycteris minuta|[38]|Guyana.Potaro-Siparuni|Phyllostomidae|
Micronycteris minuta|[39]|Guyana.East Berbice-Corentyne|Phyllostomidae|
Micronycteris minuta|[40]|Guyana.Demerara-Mahaica|Phyllostomidae|
Micronycteris minuta|[41]|Guyana.Potaro-Siparuni|Phyllostomidae|
Micronycteris cf. minuta|[42]|French Guiana.Cayenne|Phyllostomidae|
Micronycteris minuta|[43]|Ecuador.Napo|Phyllostomidae|
Micronycteris minuta|[44]|Ecuador.Napo|Phyllostomidae|
Micronycteris minuta|[45]|Ecuador.Napo|Phyllostomidae|
Micronycteris minuta|[46]|Ecuador.Napo|Phyllostomidae|
Micronycteris minuta|[47]|Ecuador.Napo|Phyllostomidae|
Micronycteris minuta|[48]|Ecuador|Phyllostomidae|
Micronycteris minuta|[49]|Bolivia.Santa Cruz|Phyllostomidae|
Unknown Specimen||
Micronycteris sanborni|[50]|Brazil.Mato Grosso|Phyllostomidae|
Micronycteris sp.|[51]|Costa Rica.Guanacaste|Phyllostomidae|
Micronycteris sp.|[52]|Costa Rica.Guanacaste|Phyllostomidae|
Micronycteris hirsuta|[53]|Panama|Phyllostomidae|
Micronycteris megalotis|[54]|Guyana.Demerara-Mahaica|Phyllostomidae|
Micronycteris megalotis|[55]|Guyana.Potaro-Siparuni|Phyllostomidae|
Micronycteris megalotis|[56]|Suriname.Sipaliwini|Phyllostomidae|
Micronycteris megalotis|[57]|Suriname.Sipaliwini|Phyllostomidae|
Micronycteris minuta|[58]|Suriname.Sipaliwini|Phyllostomidae|
Micronycteris megalotis|[59]|Guyana.Upper Demerara-Berbice|Phyllostomidae|
Micronycteris megalotis|[60]|Suriname.Brokopondo|Phyllostomidae|
Micronycteris megalotis|[61]|Suriname.Sipaliwini|Phyllostomidae|
Micronycteris megalotis|[62]|Guyana.Potaro-Siparuni|Phyllostomidae|
Micronycteris megalotis|[63]|Guyana.Potaro-Siparuni|Phyllostomidae|
Micronycteris megalotis|[64]|Guyana.Cuyuni-Mazaruni|Phyllostomidae|
Micronycteris schmidtorum|[65]|Mexico.Campeche|Phyllostomidae|
Micronycteris minuta|[66]|Mexico.Campeche|Phyllostomidae|
Artibeus concolor|[67]|Suriname.Sipaliwini|Phyllostomidae|
Sturnira ludovici|[68]|Honduras.Francisco Morazan|Phyllostomidae|
Sturnira ludovici|[69]|El Salvador.Santa Ana|Phyllostomidae|
Sturnira ludovici|[70]|El Salvador.Santa Ana|Phyllostomidae|
Sturnira ludovici|[71]|El Salvador.Santa Ana|Phyllostomidae|
Sturnira ludovici|[72]|El Salvador.Santa Ana|Phyllostomidae|
Sturnira ludovici|[73]|Honduras.Francisco Morazan|Phyllostomidae|
Sturnira ludovici|[74]|Honduras.Francisco Morazan|Phyllostomidae|
Sturnira ludovici|[75]|El Salvador.Santa Ana|Phyllostomidae|
Sturnira ludovici|[76]|El Salvador.Santa Ana|Phyllostomidae|
Sturnira ludovici|[77]|El Salvador.Santa Ana|Phyllostomidae|
Sturnira ludovici|[78]|El Salvador.Santa Ana|Phyllostomidae|
Sturnira ludovici|[79]|Guatemala.El Progreso|Phyllostomidae|
Sturnira ludovici|[80]|El Salvador.Santa Ana|Phyllostomidae|
Sturnira ludovici|[81]|Honduras.Francisco Morazan|Phyllostomidae|
Sturnira ludovici|[82]|Mexico.Chiapas|Phyllostomidae|
Sturnira ludovici|[83]|Mexico.Chiapas|Phyllostomidae|
Sturnira bidens|[84]|Ecuador.Napo|Phyllostomidae|
Rhinophylla pumilio|[85]|Guyana.Demerara-Mahaica|Phyllostomidae|
Rhinophylla pumilio|[86]|Guyana.Demerara-Mahaica|Phyllostomidae|
Rhinophylla pumilio|[87]|Guyana.Demerara-Mahaica|Phyllostomidae|
Rhinophylla pumilio|[88]|Ecuador.Napo|Phyllostomidae|
Rhinophylla pumilio|[89]|Suriname.Sipaliwini|Phyllostomidae|
Mimon cozumelae|[90]|Belize|Phyllostomidae|
Mimon cozumelae|[91]|Mexico.Yucatan|Phyllostomidae|
Mimon cozumelae|[92]|Mexico.Campeche|Phyllostomidae|
Mimon cozumelae|[93]|Mexico.Yucatan|Phyllostomidae|
Mimon cozumelae|[94]|Guatemala.Peten|Phyllostomidae|
Mimon cozumelae|[95]|Mexico.Campeche|Phyllostomidae|
Mimon cozumelae|[96]|Mexico.Campeche|Phyllostomidae|
Carollia perspicillata|[97]|Guyana.Potaro-Siparuni|Phyllostomidae|
Carollia perspicillata|[98]|Brazil.Roraima|Phyllostomidae|
Carollia perspicillata|[99]|Suriname.Nickerie|Phyllostomidae|
