# Supplementary material for: Using DNA Barcodes to Identify Road-Killed Animals in Two Atlantic Forest Nature Reserves, Brazil
Source: PLoS One. 2015 Aug 5;10(8):e0134877. doi: 10.1371/journal.pone.0134877 (PMC4526655; doi:10.1371/journal.pone.0134877)
Supplement: S6 Fig — (PDF) [file pone.0134877.s006.pdf]

BOLD TaxonID Tree

```
Title            : COI SPECIES DATABASE Tree
Date             : 16-June-2015
Data Type        : Nucleotide
Distance Model   : Kimura 2 Parameter
Marker           : COI-5P
Codon Positions  : 1st, 2nd, 3rd
Labels           : Extra Info, Country & Province, Family
Filters          : Length > 200
Attachment       : Photographs & Spreadsheet

Sequence Count   : 100
Species count    : 6
Genus count      : 3
Family count     : 1
Unidentified     : 1
```

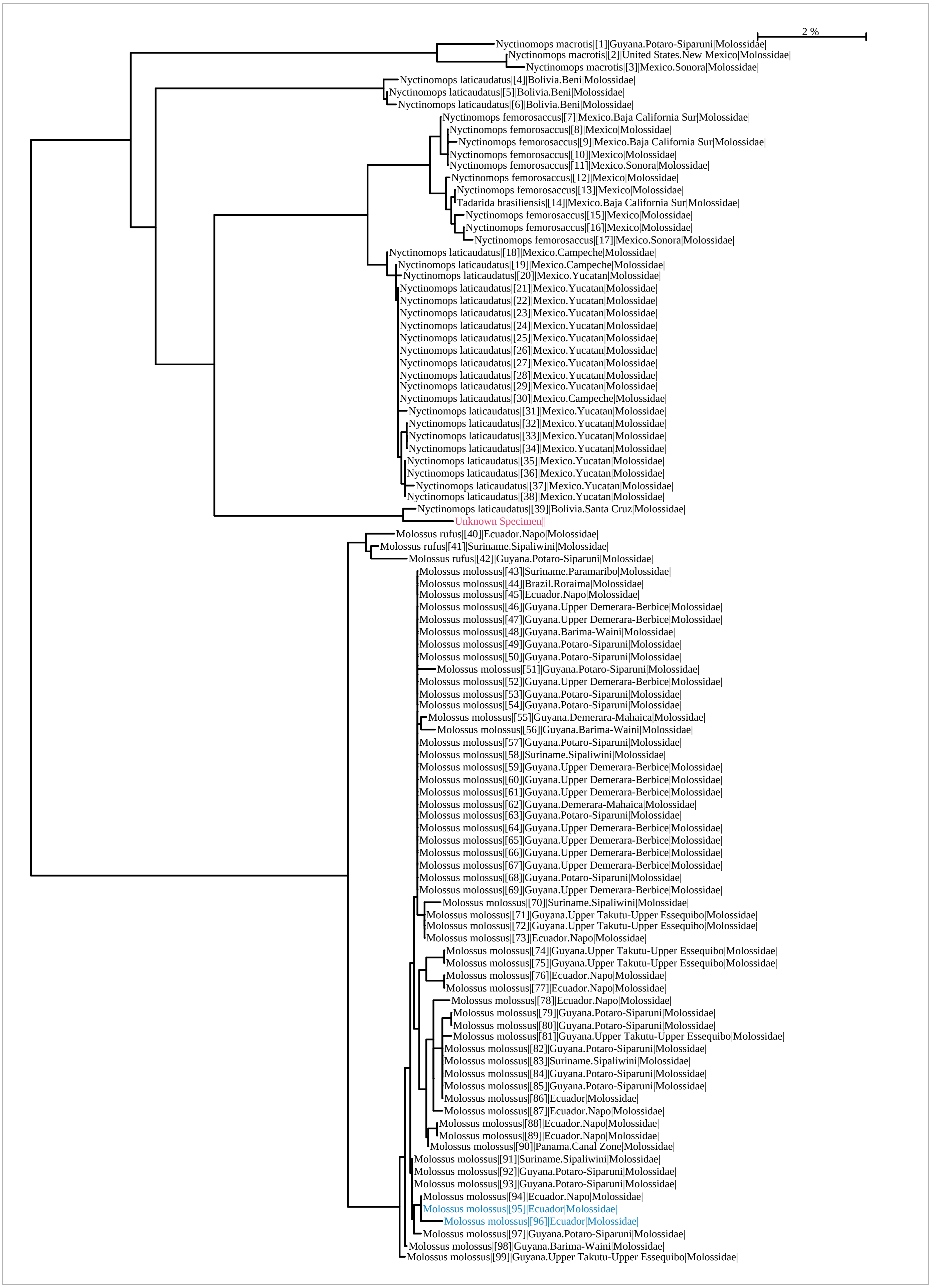
2 %
Nyctinomops macrotis|[1]|Guyana.Potaro-Siparuni|Molossidae|
Nyctinomops macrotis|[2]|United States.New Mexico|Molossidae|
Nyctinomops macrotis|[3]|Mexico.Sonora|Molossidae|
Nyctinomops laticaudatus|[4]|Bolivia.Beni|Molossidae|
Nyctinomops laticaudatus|[5]|Bolivia.Beni|Molossidae|
Nyctinomops laticaudatus|[6]|Bolivia.Beni|Molossidae|
Nyctinomops femorosaccus|[7]|Mexico.Baja California Sur|Molossidae|
Nyctinomops femorosaccus|[8]|Mexico|Molossidae|
Nyctinomops femorosaccus|[9]|Mexico.Baja California Sur|Molossidae|
Nyctinomops femorosaccus|[10]|Mexico|Molossidae|
Nyctinomops femorosaccus|[11]|Mexico.Sonora|Molossidae|
Nyctinomops femorosaccus|[12]|Mexico|Molossidae|
Nyctinomops femorosaccus|[13]|Mexico|Molossidae|
Tadarida brasiliensis|[14]|Mexico.Baja California Sur|Molossidae|
Nyctinomops femorosaccus|[15]|Mexico|Molossidae|
Nyctinomops femorosaccus|[16]|Mexico|Molossidae|
Nyctinomops femorosaccus|[17]|Mexico.Sonora|Molossidae|
Nyctinomops laticaudatus|[18]|Mexico.Campeche|Molossidae|
Nyctinomops laticaudatus|[19]|Mexico.Campeche|Molossidae|
Nyctinomops laticaudatus|[20]|Mexico.Yucatan|Molossidae|
Nyctinomops laticaudatus|[21]|Mexico.Yucatan|Molossidae|
Nyctinomops laticaudatus|[22]|Mexico.Yucatan|Molossidae|
Nyctinomops laticaudatus|[23]|Mexico.Yucatan|Molossidae|
Nyctinomops laticaudatus|[24]|Mexico.Yucatan|Molossidae|
Nyctinomops laticaudatus|[25]|Mexico.Yucatan|Molossidae|
Nyctinomops laticaudatus|[26]|Mexico.Yucatan|Molossidae|
Nyctinomops laticaudatus|[27]|Mexico.Yucatan|Molossidae|
Nyctinomops laticaudatus|[28]|Mexico.Yucatan|Molossidae|
Nyctinomops laticaudatus|[29]|Mexico.Yucatan|Molossidae|
Nyctinomops laticaudatus|[30]|Mexico.Campeche|Molossidae|
Nyctinomops laticaudatus|[31]|Mexico.Yucatan|Molossidae|
Nyctinomops laticaudatus|[32]|Mexico.Yucatan|Molossidae|
Nyctinomops laticaudatus|[33]|Mexico.Yucatan|Molossidae|
Nyctinomops laticaudatus|[34]|Mexico.Yucatan|Molossidae|
Nyctinomops laticaudatus|[35]|Mexico.Yucatan|Molossidae|
Nyctinomops laticaudatus|[36]|Mexico.Yucatan|Molossidae|
Nyctinomops laticaudatus|[37]|Mexico.Yucatan|Molossidae|
Nyctinomops laticaudatus|[38]|Mexico.Yucatan|Molossidae|
Nyctinomops laticaudatus|[39]|Bolivia.Santa Cruz|Molossidae|
Unknown Specimen||
Molossus rufus|[40]|Ecuador.Napo|Molossidae|
Molossus rufus|[41]|Suriname.Sipaliwini|Molossidae|
Molossus rufus|[42]|Guyana.Potaro-Siparuni|Molossidae|
Molossus molossus|[43]|Suriname.Paramaribo|Molossidae|
Molossus molossus|[44]|Brazil.Roraima|Molossidae|
Molossus molossus|[45]|Ecuador.Napo|Molossidae|
Molossus molossus|[46]|Guyana.Upper Demerara-Berbice|Molossidae|
Molossus molossus|[47]|Guyana.Upper Demerara-Berbice|Molossidae|
Molossus molossus|[48]|Guyana.Barima-Waini|Molossidae|
Molossus molossus|[49]|Guyana.Potaro-Siparuni|Molossidae|
Molossus molossus|[50]|Guyana.Potaro-Siparuni|Molossidae|
Molossus molossus|[51]|Guyana.Potaro-Siparuni|Molossidae|
Molossus molossus|[52]|Guyana.Upper Demerara-Berbice|Molossidae|
Molossus molossus|[53]|Guyana.Potaro-Siparuni|Molossidae|
Molossus molossus|[54]|Guyana.Potaro-Siparuni|Molossidae|
Molossus molossus|[55]|Guyana.Demerara-Mahaica|Molossidae|
Molossus molossus|[56]|Guyana.Barima-Waini|Molossidae|
Molossus molossus|[57]|Guyana.Potaro-Siparuni|Molossidae|
Molossus molossus|[58]|Suriname.Sipaliwini|Molossidae|
Molossus molossus|[59]|Guyana.Upper Demerara-Berbice|Molossidae|
Molossus molossus|[60]|Guyana.Upper Demerara-Berbice|Molossidae|
Molossus molossus|[61]|Guyana.Upper Demerara-Berbice|Molossidae|
Molossus molossus|[62]|Guyana.Demerara-Mahaica|Molossidae|
Molossus molossus|[63]|Guyana.Potaro-Siparuni|Molossidae|
Molossus molossus|[64]|Guyana.Upper Demerara-Berbice|Molossidae|
Molossus molossus|[65]|Guyana.Upper Demerara-Berbice|Molossidae|
Molossus molossus|[66]|Guyana.Upper Demerara-Berbice|Molossidae|
Molossus molossus|[67]|Guyana.Upper Demerara-Berbice|Molossidae|
Molossus molossus|[68]|Guyana.Potaro-Siparuni|Molossidae|
Molossus molossus|[69]|Guyana.Upper Demerara-Berbice|Molossidae|
Molossus molossus|[70]|Suriname.Sipaliwini|Molossidae|
Molossus molossus|[71]|Guyana.Upper Takutu-Upper Essequibo|Molossidae|
Molossus molossus|[72]|Guyana.Upper Takutu-Upper Essequibo|Molossidae|
Molossus molossus|[73]|Ecuador.Napo|Molossidae|
Molossus molossus|[74]|Guyana.Upper Takutu-Upper Essequibo|Molossidae|
Molossus molossus|[75]|Guyana.Upper Takutu-Upper Essequibo|Molossidae|
Molossus molossus|[76]|Ecuador.Napo|Molossidae|
Molossus molossus|[77]|Ecuador.Napo|Molossidae|
Molossus molossus|[78]|Ecuador.Napo|Molossidae|
Molossus molossus|[79]|Guyana.Potaro-Siparuni|Molossidae|
Molossus molossus|[80]|Guyana.Potaro-Siparuni|Molossidae|
Molossus molossus|[81]|Guyana.Upper Takutu-Upper Essequibo|Molossidae|
Molossus molossus|[82]|Guyana.Potaro-Siparuni|Molossidae|
Molossus molossus|[83]|Suriname.Sipaliwini|Molossidae|
Molossus molossus|[84]|Guyana.Potaro-Siparuni|Molossidae|
Molossus molossus|[85]|Guyana.Potaro-Siparuni|Molossidae|
Molossus molossus|[86]|Ecuador|Molossidae|
Molossus molossus|[87]|Ecuador.Napo|Molossidae|
Molossus molossus|[88]|Ecuador.Napo|Molossidae|
Molossus molossus|[89]|Ecuador.Napo|Molossidae|
Molossus molossus|[90]|Panama.Canal Zone|Molossidae|
Molossus molossus|[91]|Suriname.Sipaliwini|Molossidae|
Molossus molossus|[92]|Guyana.Potaro-Siparuni|Molossidae|
Molossus molossus|[93]|Guyana.Potaro-Siparuni|Molossidae|
Molossus molossus|[94]|Ecuador.Napo|Molossidae|
Molossus molossus|[95]|Ecuador|Molossidae|
Molossus molossus|[96]|Ecuador|Molossidae|
Molossus molossus|[97]|Guyana.Potaro-Siparuni|Molossidae|
Molossus molossus|[98]|Guyana.Barima-Waini|Molossidae|
Molossus molossus|[99]|Guyana.Upper Takutu-Upper Essequibo|Molossidae|
