# Supplementary material for: Using DNA Barcodes to Identify Road-Killed Animals in Two Atlantic Forest Nature Reserves, Brazil
Source: PLoS One. 2015 Aug 5;10(8):e0134877. doi: 10.1371/journal.pone.0134877 (PMC4526655; doi:10.1371/journal.pone.0134877)

BOLD TaxonID Tree

Title : COI SPECIES DATABASE Tree
Date : 16-June-2015
Data Type : Nucleotide
Distance Model : Kimura 2 Parameter
Marker : COI-5P
Codon Positions : 1st, 2nd, 3rd
Labels : Extra Info, Country & Province, Family
Filters : Length > 200
Attachment : Photographs & Spreadsheet

Sequence Count : 100
Species count : 4
Genus count : 2
Family count : 1
Unidentified : 1

COI_SPECIES_DATABASE_Tree Tue Jun 16 14:43:02 2015 Page 1 of 1

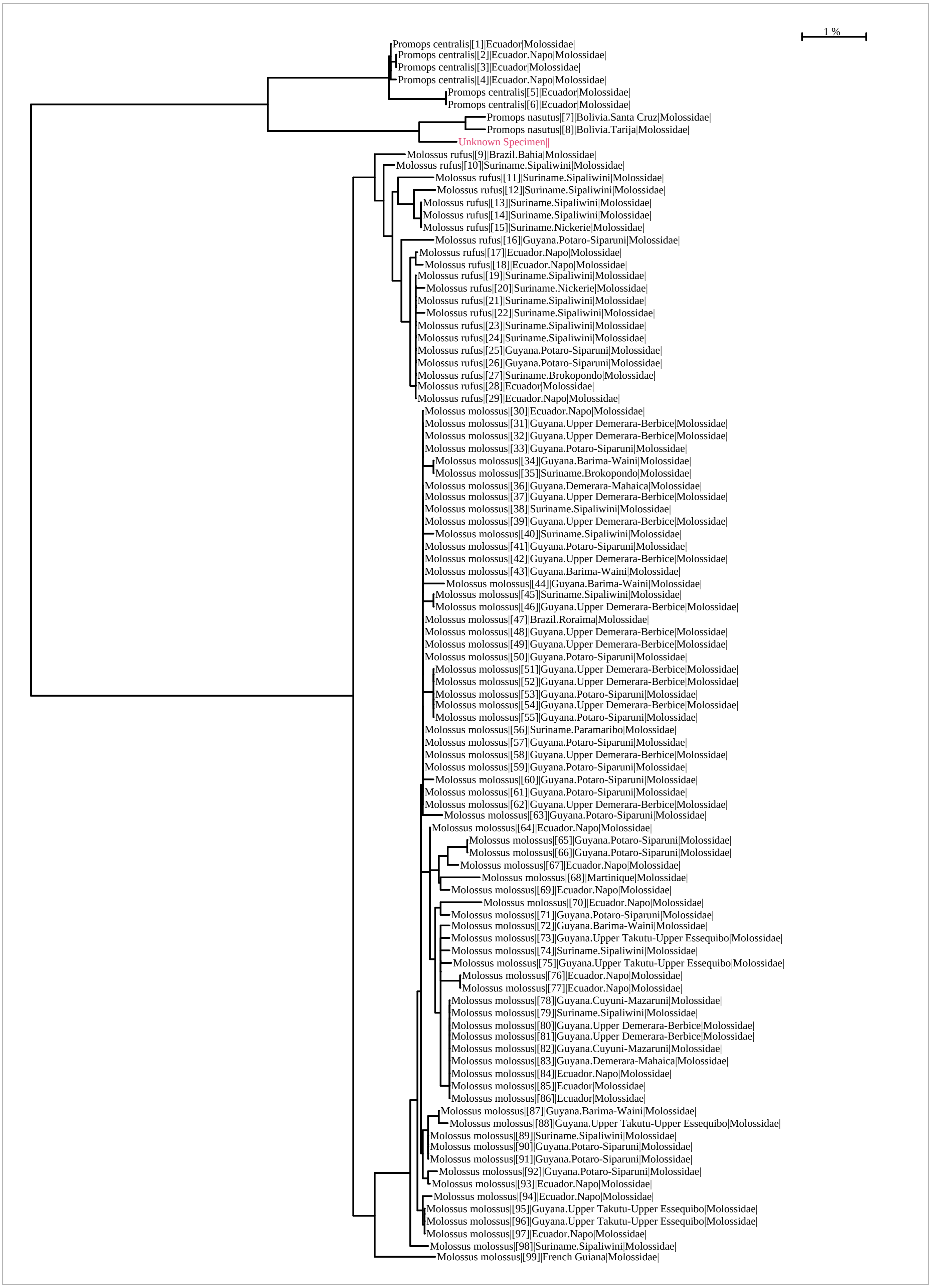

Supplement: S8 Fig — (PDF) [file pone.0134877.s008.pdf]
