# Supplementary material for: Using DNA Barcodes to Identify Road-Killed Animals in Two Atlantic Forest Nature Reserves, Brazil
Source: PLoS One. 2015 Aug 5;10(8):e0134877. doi: 10.1371/journal.pone.0134877 (PMC4526655; doi:10.1371/journal.pone.0134877)
Supplement: S9 Fig — (PDF) [file pone.0134877.s009.pdf]

BOLD TaxonID Tree

Title	:	COI SPECIES DATABASE Tree
Date	:	16-June-2015
Data Type	:	Nucleotide
Distance Model	:	Kimura 2 Parameter
Marker	:	COI-5P
Codon Positions	:	1st, 2nd, 3rd
Labels	:	Extra Info, Country & Province, Family
Filters	:	Length > 200
Attachment	:	Photographs & Spreadsheet

Sequence Count	:	100
Species count	:	31
Genus count	:	16
Family count	:	4
Unidentified	:	1

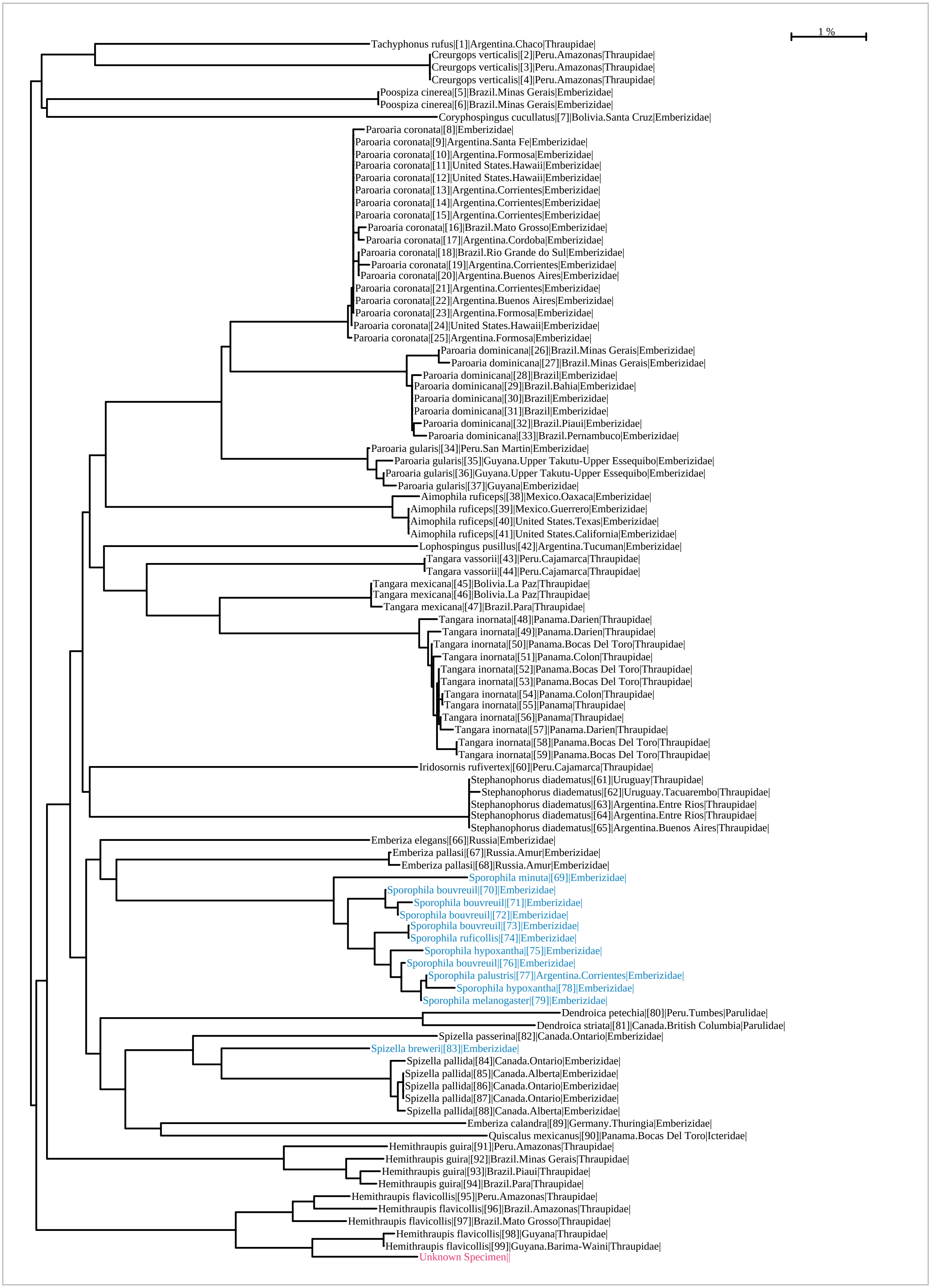
1 %
Tachyphonus rufus|[1]|Argentina.Chaco|Thraupidae|
Creurgops verticalis|[2]|Peru.Amazonas|Thraupidae|
Creurgops verticalis|[3]|Peru.Amazonas|Thraupidae|
Creurgops verticalis|[4]|Peru.Amazonas|Thraupidae|
Poospiza cinerea|[5]|Brazil.Minas Gerais|Emberizidae|
Poospiza cinerea|[6]|Brazil.Minas Gerais|Emberizidae|
Coryphospingus cucullatus|[7]|Bolivia.Santa Cruz|Emberizidae|
Paroaria coronata|[8]|Emberizidae|
Paroaria coronata|[9]|Argentina.Santa Fe|Emberizidae|
Paroaria coronata|[10]|Argentina.Formosa|Emberizidae|
Paroaria coronata|[11]|United States.Hawaii|Emberizidae|
Paroaria coronata|[12]|United States.Hawaii|Emberizidae|
Paroaria coronata|[13]|Argentina.Corrientes|Emberizidae|
Paroaria coronata|[14]|Argentina.Corrientes|Emberizidae|
Paroaria coronata|[15]|Argentina.Corrientes|Emberizidae|
Paroaria coronata|[16]|Brazil.Mato Grosso|Emberizidae|
Paroaria coronata|[17]|Argentina.Cordoba|Emberizidae|
Paroaria coronata|[18]|Brazil.Rio Grande do Sul|Emberizidae|
Paroaria coronata|[19]|Argentina.Corrientes|Emberizidae|
Paroaria coronata|[20]|Argentina.Buenos Aires|Emberizidae|
Paroaria coronata|[21]|Argentina.Corrientes|Emberizidae|
Paroaria coronata|[22]|Argentina.Buenos Aires|Emberizidae|
Paroaria coronata|[23]|Argentina.Formosa|Emberizidae|
Paroaria coronata|[24]|United States.Hawaii|Emberizidae|
Paroaria coronata|[25]|Argentina.Formosa|Emberizidae|
Paroaria dominicana|[26]|Brazil.Minas Gerais|Emberizidae|
Paroaria dominicana|[27]|Brazil.Minas Gerais|Emberizidae|
Paroaria dominicana|[28]|Brazil|Emberizidae|
Paroaria dominicana|[29]|Brazil.Bahia|Emberizidae|
Paroaria dominicana|[30]|Brazil|Emberizidae|
Paroaria dominicana|[31]|Brazil|Emberizidae|
Paroaria dominicana|[32]|Brazil.Piaui|Emberizidae|
Paroaria dominicana|[33]|Brazil.Pernambuco|Emberizidae|
Paroaria gularis|[34]|Peru.San Martin|Emberizidae|
Paroaria gularis|[35]|Guyana.Upper Takutu-Upper Essequibo|Emberizidae|
Paroaria gularis|[36]|Guyana.Upper Takutu-Upper Essequibo|Emberizidae|
Paroaria gularis|[37]|Guyana|Emberizidae|
Aimophila ruficeps|[38]|Mexico.Oaxaca|Emberizidae|
Aimophila ruficeps|[39]|Mexico.Guerrero|Emberizidae|
Aimophila ruficeps|[40]|United States.Texas|Emberizidae|
Aimophila ruficeps|[41]|United States.California|Emberizidae|
Lophospingus pusillus|[42]|Argentina.Tucuman|Emberizidae|
Tangara vassorii|[43]|Peru.Cajamarca|Thraupidae|
Tangara vassorii|[44]|Peru.Cajamarca|Thraupidae|
Tangara mexicana|[45]|Bolivia.La Paz|Thraupidae|
Tangara mexicana|[46]|Bolivia.La Paz|Thraupidae|
Tangara mexicana|[47]|Brazil.Para|Thraupidae|
Tangara inornata|[48]|Panama.Darien|Thraupidae|
Tangara inornata|[49]|Panama.Darien|Thraupidae|
Tangara inornata|[50]|Panama.Bocas Del Toro|Thraupidae|
Tangara inornata|[51]|Panama.Colon|Thraupidae|
Tangara inornata|[52]|Panama.Bocas Del Toro|Thraupidae|
Tangara inornata|[53]|Panama.Bocas Del Toro|Thraupidae|
Tangara inornata|[54]|Panama.Colon|Thraupidae|
Tangara inornata|[55]|Panama|Thraupidae|
Tangara inornata|[56]|Panama|Thraupidae|
Tangara inornata|[57]|Panama.Darien|Thraupidae|
Tangara inornata|[58]|Panama.Bocas Del Toro|Thraupidae|
Tangara inornata|[59]|Panama.Bocas Del Toro|Thraupidae|
Iridosornis rufivertex|[60]|Peru.Cajamarca|Thraupidae|
Stephanophorus diadematus|[61]|Uruguay|Thraupidae|
Stephanophorus diadematus|[62]|Uruguay.Tacuarembo|Thraupidae|
Stephanophorus diadematus|[63]|Argentina.Entre Rios|Thraupidae|
Stephanophorus diadematus|[64]|Argentina.Entre Rios|Thraupidae|
Stephanophorus diadematus|[65]|Argentina.Buenos Aires|Thraupidae|
Emberiza elegans|[66]|Russia|Emberizidae|
Emberiza pallasi|[67]|Russia.Amur|Emberizidae|
Emberiza pallasi|[68]|Russia.Amur|Emberizidae|
Sporophila minuta|[69]|Emberizidae|
Sporophila bouvreuil|[70]|Emberizidae|
Sporophila bouvreuil|[71]|Emberizidae|
Sporophila bouvreuil|[72]|Emberizidae|
Sporophila bouvreuil|[73]|Emberizidae|
Sporophila ruficollis|[74]|Emberizidae|
Sporophila hypoxantha|[75]|Emberizidae|
Sporophila bouvreuil|[76]|Emberizidae|
Sporophila palustris|[77]|Argentina.Corrientes|Emberizidae|
Sporophila hypoxantha|[78]|Emberizidae|
Sporophila melanogaster|[79]|Emberizidae|
Dendroica petechia|[80]|Peru.Tumbes|Parulidae|
Dendroica striata|[81]|Canada.British Columbia|Parulidae|
Spizella passerina|[82]|Canada.Ontario|Emberizidae|
Spizella breweri|[83]|Emberizidae|
Spizella pallida|[84]|Canada.Ontario|Emberizidae|
Spizella pallida|[85]|Canada.Alberta|Emberizidae|
Spizella pallida|[86]|Canada.Ontario|Emberizidae|
Spizella pallida|[87]|Canada.Ontario|Emberizidae|
Spizella pallida|[88]|Canada.Alberta|Emberizidae|
Emberiza calandra|[89]|Germany.Thuringia|Emberizidae|
Quiscalus mexicanus|[90]|Panama.Bocas Del Toro|Icteridae|
Hemithraupis guira|[91]|Peru.Amazonas|Thraupidae|
Hemithraupis guira|[92]|Brazil.Minas Gerais|Thraupidae|
Hemithraupis guira|[93]|Brazil.Piaui|Thraupidae|
Hemithraupis guira|[94]|Brazil.Para|Thraupidae|
Hemithraupis flavicollis|[95]|Peru.Amazonas|Thraupidae|
Hemithraupis flavicollis|[96]|Brazil.Amazonas|Thraupidae|
Hemithraupis flavicollis|[97]|Brazil.Mato Grosso|Thraupidae|
Hemithraupis flavicollis|[98]|Guyana|Thraupidae|
Hemithraupis flavicollis|[99]|Guyana.Barima-Waini|Thraupidae|
Unknown Specimen||
