# Supplementary material for: Using DNA Barcodes to Identify Road-Killed Animals in Two Atlantic Forest Nature Reserves, Brazil
Source: PLoS One. 2015 Aug 5;10(8):e0134877. doi: 10.1371/journal.pone.0134877 (PMC4526655; doi:10.1371/journal.pone.0134877)
Supplement: S10 Fig — (PDF) [file pone.0134877.s010.pdf]

BOLD TaxonID Tree

Title	:	COI SPECIES DATABASE Tree
Date	:	16-June-2015
Data Type	:	Nucleotide
Distance Model	:	Kimura 2 Parameter
Marker	:	COI-5P
Codon Positions	:	1st, 2nd, 3rd
Labels	:	Extra Info, Country & Province, Family
Filters	:	Length > 200
Attachment	:	Photographs & Spreadsheet

Sequence Count	:	100
Species count	:	19
Genus count	:	8
Family count	:	1
Unidentified	:	1

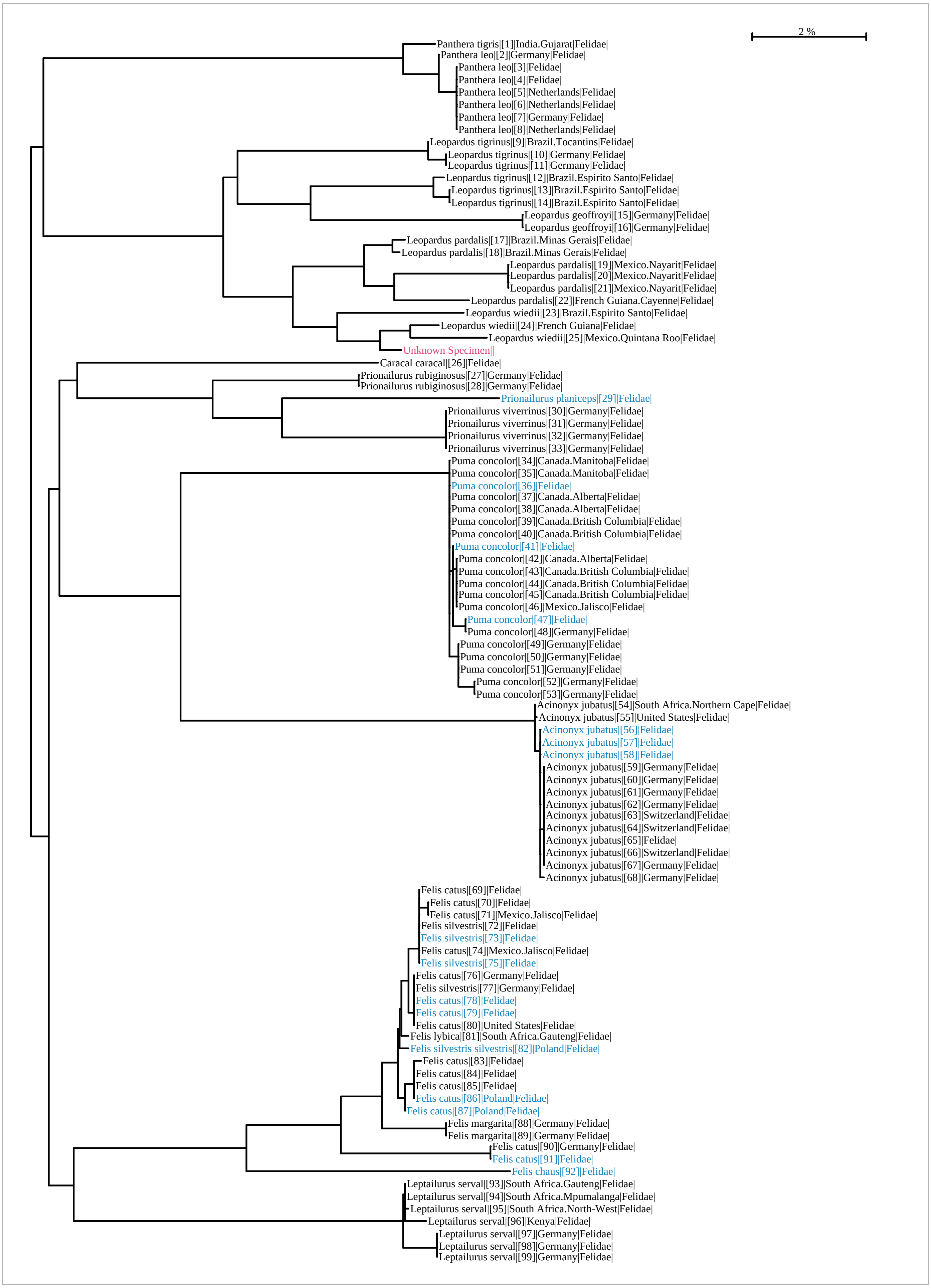
2 %
Panthera tigris|[1]|India.Gujarat|Felidae|
Panthera leo|[2]|Germany|Felidae|
Panthera leo|[3]|Felidae|
Panthera leo|[4]|Felidae|
Panthera leo|[5]|Netherlands|Felidae|
Panthera leo|[6]|Netherlands|Felidae|
Panthera leo|[7]|Germany|Felidae|
Panthera leo|[8]|Netherlands|Felidae|
Leopardus tigrinus|[9]|Brazil.Tocantins|Felidae|
Leopardus tigrinus|[10]|Germany|Felidae|
Leopardus tigrinus|[11]|Germany|Felidae|
Leopardus tigrinus|[12]|Brazil.Espirito Santo|Felidae|
Leopardus tigrinus|[13]|Brazil.Espirito Santo|Felidae|
Leopardus tigrinus|[14]|Brazil.Espirito Santo|Felidae|
Leopardus geoffroyi|[15]|Germany|Felidae|
Leopardus geoffroyi|[16]|Germany|Felidae|
Leopardus pardalis|[17]|Brazil.Minas Gerais|Felidae|
Leopardus pardalis|[18]|Brazil.Minas Gerais|Felidae|
Leopardus pardalis|[19]|Mexico.Nayarit|Felidae|
Leopardus pardalis|[20]|Mexico.Nayarit|Felidae|
Leopardus pardalis|[21]|Mexico.Nayarit|Felidae|
Leopardus pardalis|[22]|French Guiana.Cayenne|Felidae|
Leopardus wiedii|[23]|Brazil.Espirito Santo|Felidae|
Leopardus wiedii|[24]|French Guiana|Felidae|
Leopardus wiedii|[25]|Mexico.Quintana Roo|Felidae|
Unknown Specimen||
Caracal caracal|[26]|Felidae|
Prionailurus rubiginosus|[27]|Germany|Felidae|
Prionailurus rubiginosus|[28]|Germany|Felidae|
Prionailurus planiceps|[29]|Felidae|
Prionailurus viverrinus|[30]|Germany|Felidae|
Prionailurus viverrinus|[31]|Germany|Felidae|
Prionailurus viverrinus|[32]|Germany|Felidae|
Prionailurus viverrinus|[33]|Germany|Felidae|
Puma concolor|[34]|Canada.Manitoba|Felidae|
Puma concolor|[35]|Canada.Manitoba|Felidae|
Puma concolor|[36]|Felidae|
Puma concolor|[37]|Canada.Alberta|Felidae|
Puma concolor|[38]|Canada.Alberta|Felidae|
Puma concolor|[39]|Canada.British Columbia|Felidae|
Puma concolor|[40]|Canada.British Columbia|Felidae|
Puma concolor|[41]|Felidae|
Puma concolor|[42]|Canada.Alberta|Felidae|
Puma concolor|[43]|Canada.British Columbia|Felidae|
Puma concolor|[44]|Canada.British Columbia|Felidae|
Puma concolor|[45]|Canada.British Columbia|Felidae|
Puma concolor|[46]|Mexico.Jalisco|Felidae|
Puma concolor|[47]|Felidae|
Puma concolor|[48]|Germany|Felidae|
Puma concolor|[49]|Germany|Felidae|
Puma concolor|[50]|Germany|Felidae|
Puma concolor|[51]|Germany|Felidae|
Puma concolor|[52]|Germany|Felidae|
Puma concolor|[53]|Germany|Felidae|
Acinonyx jubatus|[54]|South Africa.Northern Cape|Felidae|
Acinonyx jubatus|[55]|United States|Felidae|
Acinonyx jubatus|[56]|Felidae|
Acinonyx jubatus|[57]|Felidae|
Acinonyx jubatus|[58]|Felidae|
Acinonyx jubatus|[59]|Germany|Felidae|
Acinonyx jubatus|[60]|Germany|Felidae|
Acinonyx jubatus|[61]|Germany|Felidae|
Acinonyx jubatus|[62]|Germany|Felidae|
Acinonyx jubatus|[63]|Switzerland|Felidae|
Acinonyx jubatus|[64]|Switzerland|Felidae|
Acinonyx jubatus|[65]|Felidae|
Acinonyx jubatus|[66]|Switzerland|Felidae|
Acinonyx jubatus|[67]|Germany|Felidae|
Acinonyx jubatus|[68]|Germany|Felidae|
Felis catus|[69]|Felidae|
Felis catus|[70]|Felidae|
Felis catus|[71]|Mexico.Jalisco|Felidae|
Felis silvestris|[72]|Felidae|
Felis silvestris|[73]|Felidae|
Felis catus|[74]|Mexico.Jalisco|Felidae|
Felis silvestris|[75]|Felidae|
Felis catus|[76]|Germany|Felidae|
Felis silvestris|[77]|Germany|Felidae|
Felis catus|[78]|Felidae|
Felis catus|[79]|Felidae|
Felis catus|[80]|United States|Felidae|
Felis lybica|[81]|South Africa.Gauteng|Felidae|
Felis silvestris silvestris|[82]|Poland|Felidae|
Felis catus|[83]|Felidae|
Felis catus|[84]|Felidae|
Felis catus|[85]|Felidae|
Felis catus|[86]|Poland|Felidae|
Felis catus|[87]|Poland|Felidae|
Felis margarita|[88]|Germany|Felidae|
Felis margarita|[89]|Germany|Felidae|
Felis catus|[90]|Germany|Felidae|
Felis catus|[91]|Felidae|
Felis chaus|[92]|Felidae|
Leptailurus serval|[93]|South Africa.Gauteng|Felidae|
Leptailurus serval|[94]|South Africa.Mpumalanga|Felidae|
Leptailurus serval|[95]|South Africa.North-West|Felidae|
Leptailurus serval|[96]|Kenya|Felidae|
Leptailurus serval|[97]|Germany|Felidae|
Leptailurus serval|[98]|Germany|Felidae|
Leptailurus serval|[99]|Germany|Felidae|
