# Supplementary material for: Using DNA Barcodes to Identify Road-Killed Animals in Two Atlantic Forest Nature Reserves, Brazil
Source: PLoS One. 2015 Aug 5;10(8):e0134877. doi: 10.1371/journal.pone.0134877 (PMC4526655; doi:10.1371/journal.pone.0134877)

BOLD TaxonID Tree

Title	: COI SPECIES DATABASE Tree
Date	: 25-June-2015
Data Type	: Nucleotide
Distance Model	: Kimura 2 Parameter
Marker	: COI-5P
Codon Positions	: 1st, 2nd, 3rd
Labels	: Extra Info, Country & Province, Family
Filters	: Length > 200
Attachment	: Photographs & Spreadsheet

Sequence Count	: 100
Species count	: 47
Genus count	: 21
Family count	: 1
Unidentified	: 1

COI_SPECIES_DATABASE_Tree Thu Jun 25 11:54:34 2015 Page 1 of 1

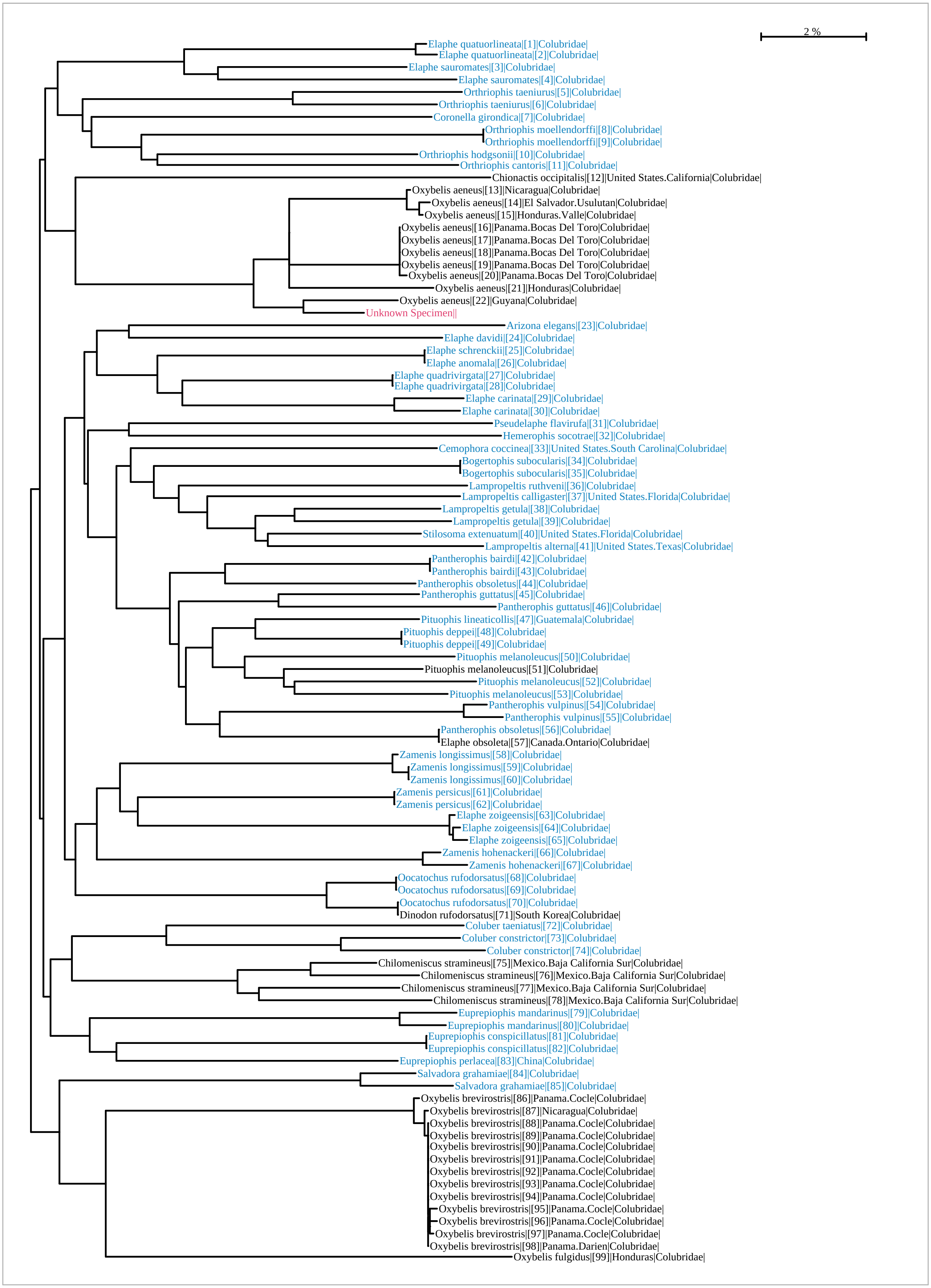

Supplement: S11 Fig — (PDF) [file pone.0134877.s011.pdf]
