# Supplementary material for: Using DNA Barcodes to Identify Road-Killed Animals in Two Atlantic Forest Nature Reserves, Brazil
Source: PLoS One. 2015 Aug 5;10(8):e0134877. doi: 10.1371/journal.pone.0134877 (PMC4526655; doi:10.1371/journal.pone.0134877)
Supplement: S1 Table — (DOC) [file pone.0134877.s012.doc]

| **Sample** | **Taxonomic group previously known** | **Species identified - DNA barcoding** | **Species identified - Photo** |
| --- | --- | --- | --- |
| 1 | Unknown | non-identified | no photo |
| 2 | Bird | *Buteo magnirostris* | non-identified |
| 3 | Bat | *Lophostoma brasiliense* | non-identified |
| 4 | Bat | non-identified | non-identified |
| 5 | Bat | *Molossus molossus* | non-identified |
| 6 | Bird | *Pipra rubrocapilla* | non-identified |
| 7 | Non-volant mammals | *Didelphis aurita* | non-identified |
| 8 | Bat | non-identified | *Saccopteryx bilineata* |
| 9 | Non-volant mammals | *Gracilinanus microtarsus* | no photo |
| 10 | Bat | *Molossus molossus* | *Molossus molossus* |
| 11 | Bat | *Saccopteryx bilineata* | non-identified |
| 12 | Bat | non-identified | non-identified |
| 13 | Bat | non-identified | *Molossus molossus* |
| 14 | Bat | non-identified | non-identified |
| 15 | Bat | *Molossus molossus* | non-identified |
| 16 | Bird | Ambiguous | non-identified |
| 17 | Bat | *Molossus molossus* | no photo |
| 18 | Non-volant mammals | *Cuniculus paca* | *Cuniculus paca* |
| 19 | Bat | non-identified | *Molossus molossus* |
| 20 | Bat | *Molossus molossus* | non-identified |
| 21 | Bird | *Euphonia violacea* | no photo |
| 22 | Bird | *Lepidocolaptes squamatus* | no photo |
| 23 | Bird | *Euphonia xanthogaster* | non-identified |
| 24 | Bat | *Carollia perspicillata* | non-identified |
| 25 | Bat | *Lampronycteris brachyotis* | no photo |
| 26 | Bat | *Micronycteris minuta* | no photo |
| 27 | Bat | *Molossus molossus* | no photo |
| 28 | Bat | non-identified | non-identified |
| 29 | Non-volant mammals | *Didelphis aurita* | *Didelphis aurita* |
| 30 | Bird | *Buteo magnirostris* | *Buteo magnirostris* |
| 31 | Bat | Ambiguous | non-identified |
| 32 | Bird | *Euphonia violacea* | non-identified |
| 33 | Bat | non-identified | *Centronycteris maximiliani* |
| 34 | Bat | non-identified | non-identified |
| 35 | Bird | Ambiguous | no photo |
| 36 | Non-volant mammals | *Cuniculus paca* | *Cuniculus paca* |
| 37 | Bat | non-identified | no photo |
| 38 | Bird | *Tangara seledon* | no photo |
| 39 | Bird | Ambiguous | non-identified |
| 40 | Bird | *Dixiphia pipra* | non-identified |
| 41 | Bat | *Vampyressa pusilla* | non-identified |
| 42 | Bat | *Molossus molossus* | no photo |
| 43 | Bird | *Myrmotherula axillaris* | non-identified |
| 44 | Bird | [*Pteroglossus aracari*](http://www.boldsystems.org/index.php/Taxbrowser_Taxonpage?taxon=Pteroglossus aracari) | no photo |
| 45 | Bat | non-identified | no photo |
| 46 | Bird | *Pyrrhura cruentata* | no photo |
| 47 | Non-volant mammals | non-identified | no photo |
| 48 | Non-volant mammals | *Cuniculus paca* | *Cuniculus paca* |
| 49 | Unknown | non-identified | no photo |
| 50 | Bat | non-identified | non-identified |
| 51 | Bat | non-identified | non-identified |
| 52 | Bat | non-identified | *Nyctinomops laticaudatus* |
| 53 | Amphibian | *Aparasphenodon brunoi* | no photo |
| 54 | Amphibian | *Trachycephalus mesophaeus* | no photo |
| 55 | Bird | *Pipra rubrocapilla* | no photo |
| 56 | Bird | *Pipra rubrocapilla* | no photo |
| 57 | Bird | non-identified | no photo |
| 58 | Bird | *Saltator maximus* | no photo |
| 59 | Reptile | non-identified | no photo |
| 60 | Amphibian | Ambiguous | no photo |
| 61 | Bird | *Coccyzus melacoryphus* | no photo |
| 62 | Bird | *Pachyramphus polychopterus* | no photo |
| 63 | Reptile | non-identified | no photo |
| 64 | Non-volant mammals | *Dasypus septemcinctus* | no photo |
| 65 | Non-volant mammals | *Didelphis aurita* | no photo |
| 66 | Non-volant mammals | *Marmosops incanus* | no photo |
| 67 | Reptile | non-identified | no photo |
| 68 | Reptile | non-identified | no photo |
| 69 | Reptile | non-identified | no photo |
| 70 | Reptile | non-identified | no photo |
| 71 | Reptile | non-identified | no photo |
| 72 | Reptile | non-identified | no photo |
| 73 | Reptile | non-identified | no photo |
| 74 | Bat | non-identified | no photo |
| 75 | Reptile | non-identified | no photo |
| 76 | Bat | *Molossus molossus* | no photo |
| 77 | Bat | non-identified | no photo |
| 78 | Non-volant mammals | *Marmosa murina* | no photo |
| 79 | Bat | non-identified | no photo |
| 80 | Bat | *Myotis riparius* | no photo |
| 81 | Amphibian | *Scinax alter* | no photo |
| 82 | Bat | *Molossus molossus* | no photo |
| 83 | Bird | *Piaya cayana* | no photo |
| 84 | Amphibian | non-identified | no photo |
| 85 | Amphibian | non-identified | no photo |
| 86 | Reptile | non-identified | no photo |
| 87 | Unknown | *Marmosops incanus* | no photo |
| 88 | Unknown | *Trachycephalus mesophaeus* | no photo |
| 89 | Unknown | *Trachycephalus mesophaeus* | no photo |
| 90 | Unknown | *Molossus molossus* | no photo |
| 91 | Unknown | *Trachycephalus mesophaeus* | no photo |
| 92 | Unknown | [*Callithrix geoffroyi*](http://www.boldsystems.org/index.php/Taxbrowser_Taxonpage?taxon=Callithrix geoffroyi) | no photo |
| 93 | Amphibian | non-identified | no photo |
| 94 | Unknown | *Trachycephalus mesophaeus* | no photo |
| 95 | Unknown | *Molossus molossus* | no photo |
| 96 | Bird | *Saltator maximus* | no photo |
| 97 | Bird | *Pipra rubrocapilla* | no photo |
| 98 | Bat | *Molossus molossus* | no photo |
| 99 | Reptile | non-identified | no photo |
| 100 | Amphibian | *Trachycephalus mesophaeus* | no photo |
| 101 | Unknown | *Molossus molossus* | no photo |
| 102 | Unknown | non-identified | no photo |
| 103 | Unknown | non-identified | no photo |
| 104 | Amphibian | *Scinax alter* | no photo |
| 105 | Unknown | non-identified | no photo |
| 106 | Unknown | non-identified | no photo |
| 107 | Unknown | non-identified | no photo |
| 108 | Unknown | non-identified | no photo |
| 109 | Unknown | *Tapera naevia* | no photo |
| 110 | Amphibian | *Trachycephalus mesophaeus* | no photo |
| 111 | Unknown | non-identified | no photo |
| 112 | Unknown | Ambiguous | no photo |
| 113 | Unknown | *Molossus molossus* | no photo |
| 114 | Unknown | *Molossus molossus* | no photo |
| 115 | Amphibian | *Trachycephalus mesophaeus* | no photo |
| 116 | Unknown | non-identified | no photo |
| 117 | Bat | *Promops nasutus* | no photo |
| 118 | Reptile | non-identified | no photo |
| 119 | Amphibian | non-identified | no photo |
| 120 | Bird | *Patagioenas picazuro* | no photo |
| 121 | Bat | *Artibeus gnomus* | no photo |
| 122 | Bird | *Myrmotherula axillaris* | no photo |
| 123 | Reptile | non-identified | no photo |
| 124 | Bird | *Pachyramphus polychopterus* | no photo |
| 125 | Reptile | non-identified | no photo |
| 126 | Bat | *Chiroderma villosum* | no photo |
| 127 | Bat | *Chiroderma villosum* | no photo |
| 128 | Non-volant mammals | *Marmosops incanus* | no photo |
| 129 | Non-volant mammals | *Callithrix geoffroyi* | *Callithrix geoffroyi* |
| 130 | Reptile | non-identified | no photo |
| 131 | Non-volant mammals | *Gracilinanus microtarsus* | no photo |
| 132 | Non-volant mammals | non-identified | *Sphiggurus insidiosus* |
| 133 | Bird | *Crotophaga ani* | no photo |
| 134 | Bird | *Myrmotherula axillaris* | no photo |
| 135 | Bat | *Molossus molossus* | no photo |
| 136 | Non-volant mammals | *Didelphis aurita* | *Didelphis aurita* |
| 137 | Bat | *Molossus molossus* | no photo |
| 138 | Non-volant mammals | ambiguous | no photo |
| 139 | Bat | non-identified | no photo |
| 140 | Amphibian | *Hypsiboas faber* | non-identified |
| 141 | Reptile | non-identified | non-identified |
| 142 | Non-volant mammals | *Didelphis aurita* | *Didelphis aurita* |
| 143 | Non-volant mammals | *Bradypus variegatus* | *Bradypus variegatus* |
| 144 | Bird | [*Pteroglossus aracari*](http://www.boldsystems.org/index.php/Taxbrowser_Taxonpage?taxon=Pteroglossus aracari) | *Pteroglossus aracari* |
| 145 | Reptile | non-identified | *Epicrates cenchria* |
| 146 | Bird | *Hemithraupis flavicollis* | non-identified |
| 147 | Bat | *Molossus rufus* | non-identified |
| 148 | Bird | *Vireo olivaceus* | non-identified |
| 149 | Reptile | non-identified | non-identified |
| 150 | Bat | *Promops nasutus* | *Promops nasutus* |
| 151 | Bird | *Dacnis cayana* | non-identified |
| 152 | Reptile | non-identified | *Typhlops brongersmianus* |
| 153 | Bat | non-identified | no photo |
| 154 | Bat | non-identified | non-identified |
| 155 | Non-volant mammals | *Callithrix geoffroyi* | *Callithrix geoffroyi* |
| 156 | Non-volant mammals | *Cuniculus paca* | *Cuniculus paca* |
| 157 | Reptile | non-identified | non-identified |
| 158 | Reptile | non-identified | *Pseudoboa nigra* |
| 159 | Bird | non-identified | *Tinamus solitarius* |
| 160 | Bat | *Saccopteryx bilineata* | no photo |
| 161 | Non-volant mammals | *Marmosa murina* | non-identified |
| 162 | Amphibian | *Trachycephalus mesophaeus* | non-identified |
| 163 | Reptile | non-identified | no photo |
| 164 | Non-volant mammals | *Chaetomys subspinosus* | no photo |
| 165 | Amphibian | non-identified | no photo |
| 166 | Amphibian | *Trachycephalus mesophaeus* | no photo |
| 167 | Amphibian | non-identified | no photo |
| 168 | Amphibian | *Aparasphenodon brunoi* | no photo |
| 169 | Amphibian | *Aparasphenodon brunoi* | no photo |
| 170 | Amphibian | *Aparasphenodon brunoi* | no photo |
| 171 | Amphibian | *Trachycephalus mesophaeus* | no photo |
| 172 | Non-volant mammals | *Cuniculus paca* | *Cuniculus paca* |
| 173 | Bat | *Trachops cirrhosus* | non-identified |
| 174 | Non-volant mammals | *Sylvilagus brasiliensis* | *Sylvilagus brasiliensis* |
| 175 | Unknown | *Didelphis aurita* | no photo |
| 176 | Non-volant mammals | *Leopardus wiedii* | no photo |
| 177 | Unknown | non-identified | no photo |
| 178 | Unknown | *Myrmotherula axillaris* | no photo |
| 179 | Unknown | non-identified | no photo |
| 180 | Unknown | *Tamandua tetradactyla* | no photo |
| 181 | Unknown | *Didelphis aurita* | no photo |
| 182 | Unknown | non-identified | no photo |
| 183 | Unknown | non-identified | no photo |
| 184 | Unknown | *Euphonia violacea* | no photo |
| 185 | Unknown | *Leopardus wiedii* | no photo |
| 186 | Unknown | *Didelphis aurita* | no photo |
| 187 | Unknown | *Oxybelis aeneus* | no photo |
| 188 | Unknown | *Molossus molossus* | no photo |
| 189 | Unknown | non-identified | no photo |
| 190 | Unknown | non-identified | no photo |
| 191 | Unknown | *Trachycephalus mesophaeus* | no photo |
| 192 | Unknown | *Leptodactylus natalensis* | no photo |
| 193 | Unknown | *Hypsiboas semilineatus* | no photo |
| 194 | Unknown | *Anoura geoffroyi* | no photo |
| 195 | Unknown | *Molossus molossus* | no photo |
| 196 | Bat | *Rhinophylla pumilio* | non-identified |
| 197 | Bird | *Cairina moschata* | non-identified |
| 198 | Reptile | non-identified | non-identified |
| 199 | Reptile | non-identified | non-identified |
| 200 | Bat | ambiguous | non-identified |
| 201 | Non-volant mammals | *Didelphis aurita* | *Didelphis aurita* |
| 202 | Reptile | non-identified | non-identified |
| 203 | Reptile | non-identified | non-identified |
| 204 | Non-volant mammals | *Cuniculus paca* | *Cuniculus paca* |
| 205 | Bird | *Megascops choliba* | *Megascops choliba* |
| 206 | Non-volant mammals | *Sylvilagus brasiliensis* | *Sylvilagus brasiliensis* |
| 207 | Bird | *Nyctidromus albicollis* | *Nyctidromus albicollis* |
| 208 | Non-volant mammals | *Cerdocyon thous* | *Cerdocyon thous* |
| 209 | Non-volant mammals | *Didelphis aurita* | *Didelphis aurita* |
| 210 | Bird | *Turdus leucomelas* | non-identified |
| 211 | Bird | *Porphyrio martinica* | *Porphyrio martinica* |
| 212 | Non-volant mammals | *Didelphis aurita* | *Didelphis aurita* |
| 213 | Reptile | non-identified | non-identified |
| 214 | Non-volant mammals | *Didelphis aurita* | *Didelphis aurita* |
| 215 | Non-volant mammals | non-identified | *Callithrix geoffroyi* |
| 216 | Non-volant mammals | *Didelphis aurita* | *Didelphis aurita* |
| 217 | Non-volant mammals | *Didelphis aurita* | *Didelphis aurita* |
| 218 | Non-volant mammals | non-identified | *Didelphis aurita* |
| 219 | Non-volant mammals | *Didelphis aurita* | *Didelphis aurita* |
| 220 | Bird | *Coragyps atratus* | *Coragyps atratus* |
| 221 | Non-volant mammals | *Didelphis aurita* | *Didelphis aurita* |
| 222 | Non-volant mammals | *Puma yagouaroundi* | *Puma yagouaroundi* |
